# Burden of respiratory syncytial virus (RSV) infection in Germany: a systematic review

**DOI:** 10.1186/s12879-024-09758-3

**Published:** 2024-08-20

**Authors:** Anahita Poshtiban, Moritz Wick, Mathieu Bangert, Oliver Damm

**Affiliations:** 1 Sanofi-Aventis Deutschland GmbH, Lützowstr. 107, 10785 Berlin, Germany; 2 Sanofi Vaccines, Lyon, France

**Keywords:** Respiratory syncytial virus, Respiratory infection, Disease burden, Bronchiolitis, Resource use and costs, Germany

## Abstract

**Background:**

Respiratory syncytial virus (RSV) is a major cause of acute lower respiratory infection and hospitalizations among infants, young children, and the elderly. This systematic literature review aimed to summarize the epidemiological and economic burden estimates of RSV infection at any age in Germany.

**Methods:**

We conducted a systematic literature search to identify full-text articles published from 2003 to 2023 and reporting data on the epidemiological or economic burden of RSV in Germany. Based on pre-specified eligibility criteria, data on incidence, rates of hospital and intensive care unit (ICU) admission, clinical manifestation, underlying conditions, seasonality, health care resource use and costs were extracted.

**Results:**

After screening 315 full-text articles, we included 42 articles in the review. The characteristics of the included studies were heterogenous regarding study population, setting, age groups and RSV-related outcome measures.

The most frequently reported epidemiological outcome measures were RSV detection rate (*n* = 33), followed by clinical manifestation (*n* = 19), seasonality (*n* = 18), and underlying conditions of RSV infection (*n* = 13). RSV detection rates were reported across heterogenous study populations, ranging from 5.2 to 55.4% in pediatric inpatient cases and from 2.9 to 14% in adult inpatient cases. All articles that reported RSV detection rates across several age groups demonstrated the highest burden in infants and young children. Few articles reported RSV-related outcome measures distinctively for the outpatient setting.

Health care resource use, such as hospital length of stay, ICU admission rate and treatment of patients with RSV infection were described in 23 articles, of which only one study quantified associated costs from 1999 to 2003 for children ≤ 3 years. In-hospital ICU admission rates varied between 3.6 and 45%, depending on population characteristics as age and underlying conditions.

**Conclusions:**

This systematic review revealed that RSV imposes substantial disease burden in infants, young children, and the elderly in Germany, whereby infants are particularly affected. To date, there has been limited exploration of the impact of RSV infection on healthy children or the elderly in Germany. Given their notably high reported burden in studies, the medical and economic RSV burden in these groups should move more into focus.

**Supplementary Information:**

The online version contains supplementary material available at 10.1186/s12879-024-09758-3.

## Background

Respiratory syncytial virus (RSV) imposes a substantial medical and economic burden on health care facilities, displaying an epidemic seasonal pattern [1–4]. The clinical spectrum of RSV infection exhibits a wide range of clinical presentations from mild courses to severe cases requiring hospitalization, intensive care unit (ICU) admission and respiratory support, such as mechanical ventilation. RSV infection can cause severe disease in infants and young children, but with repeated exposure and after young childhood, healthy older children and adults present with mostly mild, cold-like symptoms [3, 5–7]. Nearly all children experience an RSV infection by the age of 2 years [8, 9]. Globally, RSV is a prevalent pathogen and the primary cause of bronchiolitis in infants and young children [10]. A systematic literature review published in 2019 estimated that RSV caused 33.0 million acute lower respiratory infection (ALRI), 3.6 million hospitalizations, and 101,400 deaths worldwide in children under five years of age [3]. Additionally, RSV poses a significant burden of disease among adults aged 65 years and above, with an estimated 336,000 hospitalizations and 14,000 in-hospital deaths worldwide in 2015, as reported in a global systematic review and meta-analysis [11]. Besides the acute burden, RSV infection during early childhood may lead to long-term sequalae, increasing the risk of recurrent wheezing and asthma [12, 13]. In the elderly, RSV can exacerbate existing serious conditions such as COPD, asthma, and congestive heart failure [14, 15].

The primary treatment for patients hospitalized due to RSV-related ALRI is limited to supportive care, which include oxygen therapy, fluid management, and, in severe cases mechanical ventilation [9, 16]. Infants and young children with a history of prematurity or specific chronic conditions have been identified as having an increased risk of severe RSV-related ALRI [17, 18]. However, most children hospitalized for an RSV-related ALRI episode are born at full-term and are otherwise healthy [8, 19–21]. For adults, immunosenescence and several underlying chronic comorbidities make them particularly vulnerable to severe RSV infection [22, 23]. Therefore, prevention remains essential in reducing the burden of RSV infection. In 2022, nirsevimab, the first long-acting monoclonal antibody available for prevention of RSV in a broad infant population was licensed in the European Union and the United States, followed by a vaccine for elderly and maternal immunization in 2023 [24, 25]. In 2024, the German Standing Committee on Vaccination (STIKO) issued a recommendation for prophylaxis with nirsevimab for all newborns and infants to protect against severe respiratory infections caused by RSV in their first RSV season. Prior to these recent developments, the only available prophylactic option against RSV was passive immunization with palivizumab, which in the EU is only indicated for infants born at a gestational age of 35 weeks or less entering their first RSV season (aged < 6 months at the onset of the RSV season) and young children at high risk for severe RSV disease aged less than 2 years with bronchopulmonary disease (BPD) or hemodynamically significant congenital heart disease (CHD) [26]. Until early 2024, reimbursement of palivizumab for otherwise healthy preterm infants in Germany was further restricted to only those born at a gestational age of 29 weeks or less [27].

In Germany, a broad RSV surveillance at population-level is limited as only few RSV-specific outcomes are reported in the scope of national influenza surveillance, and RSV only became a notifiable disease in July 2023 [28, 29]. To synthesize all available studies on the burden of RSV in Germany, we conducted a systematic literature review. The main objective of this review was to summarize the epidemiological burden, clinical characteristics of symptomatic RSV infection, and RSV-attributable health care utilization across the entire population in Germany.

## Methods

### Search strategy

This systematic literature review was conducted following the Preferred Reporting Items for Systematic reviews and Meta-Analyses (PRISMA) guidelines [30, 31]. We developed search strategies in PubMed and Embase for articles published from 1st of January 2003 to 31st of May 2023 (Supplementary Table S1). Database searches were performed in December 2022 and updated in June 2023 to include newly published articles. Additionally, bibliography screening of articles included in this review and an explorative hand search were performed.

### Study selection

The review included studies reporting RSV epidemiological frequency measures such as incidence (RSV cases occurring in a specific population over a particular period of time) and detection rates (proportions of RSV positive cases at a given point in time), clinical characteristics of RSV infection, or related resource use and associated costs (Supplementary Table S2). Only peer-reviewed publications in English or German language were considered. Articles were excluded if they did not present data from Germany, did not differentiate RSV-specific outcomes from those of other ALRIs, or were letters or editorials. We further excluded articles with non-RSV-related study objectives, in which the quantification of RSV disease burden was restricted to detection rates only. If the exact same dataset and outcomes were presented in multiple articles, only one article was included.

Studies identified from the search were exported, and duplicate entries were removed using EndNote X9 (Clarivate, London UK, 2021). Two reviewers (A.P. and M.W.) independently screened titles and abstracts to retrieve potentially relevant papers based on predefined inclusion and exclusion criteria. Articles considered to be relevant at this stage were then independently assessed by two reviewers (A.P. and M.W.) in full-text form and reasons for exclusion were documented. Any disagreement among reviewers were resolved through discussion among A.P., O.D. and M.W. to reach consensus.

### Data extraction

Data were extracted by one reviewer (A.P. or O.D.) and cross-checked for accuracy by a second reviewer (M.W or A.P.). Data extraction encompassed author’s name and year of publication, main study characteristics (study type, sample size, population type as well as region and period of data collection), and findings related to RSV infection (incidence, detection rate, distribution of cases across age groups, seasons and other characteristics, underlying conditions, seasonality, clinical manifestation and resource use and costs). For detection rates, proportions of RSV positive cases detected via PCR or antigen testing were extracted, not accounting for the specificity and sensitivity of the test used. In studies where the RSV-positive subpopulation was clearly distinguishable, only the population characteristics of RSV-positive cases were extracted [32].

If articles reported the same dataset but presented non-overlapping outcomes, only divergent data from all articles were extracted. However, if previously published articles presented the same outcome values, these values were not extracted repeatedly from subsequent articles [32–35].

Data that were presented graphically and not described elsewhere in the respective articles were extracted using WebPlotDigitizer Version 4.6 and marked accordingly in Table S3. For articles that presented seasonality only graphically, but not explicitly stated elsewhere, season onsets and endings were defined based on observed, continuously occurring cases of RSV infection.

## Results

**Fig. 1:**
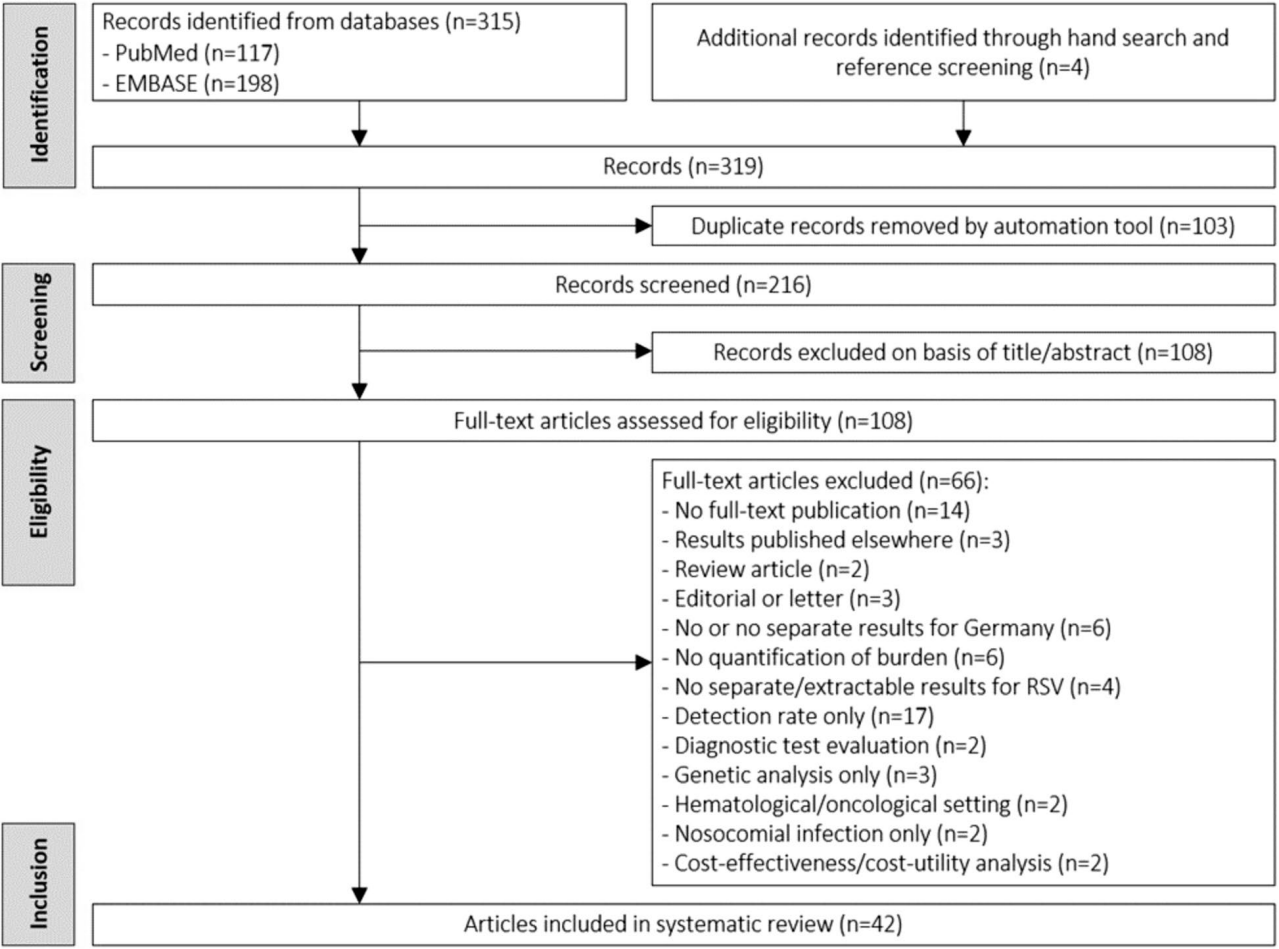
Flow diagram of the study selection process

The systematic search yielded a total of 315 articles (Fig. 1). Four additional articles were identified through the hand or bibliography search. After removing 103 duplicates, the 216 remaining articles were screened by titles and abstracts. Of these, 108 articles were selected for full-text review, ultimately resulting in 42 articles included in the review.

### Characteristics of included studies

The observation periods of the 42 included articles covered 1996 to 2023. Twenty-eight articles described multicentric or nationwide studies [17, 32, 35–60] and 14 articles single-center studies [19, 33, 34, 61–71]. Among the 42 included articles, a total of 31 unique studies were reported. Thirteen studies were classified as retrospective [17, 19, 32, 35, 38, 44, 57, 62, 64, 66–69], 22 prospective [33, 34, 37, 39, 41–43, 47–52, 54–56, 58–61, 70, 71], and the remaining did not clearly state whether the study design was retrospective or prospective. There were 2 case-control studies [32, 35] and 1 cross-sectional study [56], while no study leveraged health care claims data. Certain studies were covered by multiple articles, such as the PRI.DE-study [40–42, 71] and PID.ARI [41, 43, 57, 71].

Most articles (31/42) focused on pediatric populations [14, 19, 32–35, 40–43, 45, 47–57, 59–61, 64–68, 70, 71], while 3 focused on adults only [58, 62, 69] and the remaining articles included both children and adults [17, 36–39, 44, 63]. Three articles reported data from the outpatient setting only [36, 39, 58], 25 articles from the inpatient setting only [17, 19, 32–35, 40, 43–45, 47–49, 54, 56, 60–62, 65–71], and 14 articles from both health care setting [37, 38, 41, 42, 46, 50–53, 55, 57, 59, 63, 64]. The population sizes in the studies ranged from 214 to 413,552 subjects.

The complete list and detailed description of the studies, including main characteristics and extracted outcomes, are provided in Supplementary Table S3 and are summarized in Table 1.

**Table 1:** Summary of key study characteristics and RSV-related outcomes of articles included in the systematic literature review of RSV burden in Germany

Author	Study/population characteristics					RSV-related outcome measures									
	Population description and/or included disease/infection	Age group	Inpatient setting	Outpatient setting	Seasons	RSV detection rate (%)	RSV incidence^a^	Seasonality	Clinical manifestation	Underlying conditions	Age distribution	RSV strain differentiation	Mortality	Resource use	Costs
Alchikh et al. [61]	ILI	Pediatric	**x**		2009–2015	**x**			**x**						
Ambrosch et al. [62]	Virus-positive ARI^b^	Adult	**x**		2017–2020	**x**		**x**	**x**				**x**	**x**	
An der Heiden et al. [36]	MAARI	All ages		**x**	2010–2018	**x**	**x**				**x**				
Bierbaum et al. [37]	ARI	All ages	**x**	**x**	2009–2010	**x**		**x**							
Cai et al. [38]	ARI or ILI	All ages	**x**	**x**	2007–2017	**x**			**x**		**x**				
Cai et al. [17]	SARI	All ages	**x**		2009–2018	**x**		**x**		**x**	**x**		**x**	**x**	
Cai et al. [39]	ARI	All ages		**x**	2011–2020	**x**		**x**			**x**				
Ehlken et al. [40]	RSV-LRTI	Pediatric (≤ 3 yrs)	**x**		1999–2001										**x**
Forster [41]	LRTI	Pediatric (≤ 16 yrs)	**x**	**x**	1996–2001	**x**			**x**						
Forster et al. [42]	LRTI	Pediatric (< 3 yrs)	**x**	**x**	1999–2001	**x**	**x**		**x**			**x**		**x**	
Gröhndal et al. [43]	ALRI	Pediatric (≤ 18 yrs)	**x**		2009–2011	**x**		**x**							
Hartmann et al. [19]	RSV infection	Pediatric	**x**		2015–2018	**x**			**x**	**x**	**x**		**x**	**x**	
Hönemann et al. [63]	No specific infection	All ages	**x**	**x**	2017–2022	**x**		**x**	**x**	**x**	**x**	**x**	**x**	**x**	
Kiefer et al. [44]	RSV infection	All ages	**x**		2016–2023			**x**		**x**	**x**			**x**	
Liese et al. [45]	ARI	Pediatric (≤ 18 mo)	**x**		1998–2000	**x**	**x**		**x**	**x**	**x**			**x**	
Maison et al. [64]	Respiratory or gastrointestinal infection^c^	Pediatric (≤ 18 yrs)	**x**	**x**	2017–2021	**x**		**x**							
Mentel et al. [65]	Viral respiratory illness	Pediatric	**x**		2000–2001	**x**					**x**	**x**		**x**	
Meyer et al. [66]	Respiratory symptoms	Pediatric (≤ 4 yrs)	**x**		2020–2021	**x**								**x**	
Reiche & Schweiger [46]	Respiratory illness	All ages	**x**	**x**	1998–2007	**x**		**x**			**x**	**x**			
Schreiner et al. [67]	Pathogen-positive ALRI^d^	Pediatric	**x**		2008–2013	**x**		**x**				**x**		**x**	
Simon et al. [47]	RSV infection	Pediatric	**x**		1999–2005					**x**	**x**		**x**	**x**	
Simon et al. [48]	RSV infection	Pediatric	**x**		1999–2005					**x**	**x**		**x**	**x**	
Simon et al. [49]	Palivizumab-immunized	Pediatric (≤ 2 yrs)	**x**		2002–2007	**x**			**x**	**x**				**x**	
Simon et al. [50]	Palivizumab-immunized	Pediatric (< 25 mo)	**x**	**x**	2009–2016	**x**	**x**		**x**	**x**				**x**	
Simon et al. [51]	Palivizumab-immunized	Pediatric (< 25 mo)	**x**	**x**	2009–2016	**x**	**x**		**x**						
Simon et al. [52]	Palivizumab-immunized (incl. DS patients)	Pediatric (< 25 mo)	**x**	**x**	2009–2016	**x**	**x**			**x**					
Streng et al. [53]	RSV-RTI	Pediatric (1 mo-16 yrs)	**x**	**x**	2010–2017				**x**	**x**		**x**		**x**	
Tabatabai et al. [68]	URTI or LRTI	Pediatric (≤ 2 yrs)	**x**		2012–2013	**x**		**x**	**x**		**x**	**x**		**x**	
Tabatabai et al. [33]	ARI	Pediatric (≤ 18 yrs)	**x**		2014–2017	**x**		**x**	**x**		**x**			**x**	
Tabatabai et al. [34]	ARI	Pediatric (≤ 18 yrs)	**x**		2014–2018	**x**		**x**	**x**		**x**				
Tenenbaum et al. [54]	RSV infection	Pediatric (≤ 5 yrs)	**x**		2021–2022			**x**			**x**			**x**	
Terletskaia-Ladwig et al. [55]	Suspected RSV infection	Pediatric (≤ 18 yrs)	**x**	**x**	1996–2004	**x**		**x**			**x**				
Topoulos et al. [69]	ARI & RSV and/or influenza virus	Adult (≥ 18 yrs)	**x**	**x**	2018	**x**		**x**	**x**				**x**		
Vogel et al. [70]	ARI and/or ILI	Pediatric (≤ 18 yrs)	**x**		2009–2010	**x**		**x**	**x**		**x**	**x**		**x**	
Wasem et al. [71]	LRTI	Pediatric (≤ 36 mo)	**x**		1999–2005	**x**			**x**		**x**				
Weigl et al. [32]	LRTI	Pediatric (< 2 years)	**x**		1996–2000				**x**		**x**				
Weigl et al. [35]	LRTI	Pediatric (< 2 years)	**x**		1996–2000					**x**				**x**	
Weigl et al. [56]	CAP	Pediatric (≤ 16 yrs)	**x**		1996–2000	**x**	**x**				**x**				
Weigl et al. [57]	LRTI or URTI	Pediatric (< 16 yrs)	**x**	**x**	1996–2006	**x**		**x**			**x**				
Weinberger et al. [58]	Long-lasting cough	Adult (≥ 18 yrs)		**x**	2001–2004	**x**								**x**	
Wetzke et al. [59]	CAP	Pediatric (< 18 yrs)	**x**	**x**	2014–2020	**x**					**x**				
Wilkesmann et al. [60]	RSV infection (incl. NMI patients)	Pediatric	**x**		1999–2005					**x**	**x**		**x**	**x**	

X indicates that respective setting applies or respective outcome measure were reported

*A(L)RI* Acute (lower) respiratory infection, *CAP* Community-acquired pneumonia, *DS* Down syndrome, *ILI* Influenza-like illness, incl including, *(L)RTI* (lower) respiratory tract infection, *MAARI* Medical-attended acute respiratory infection, *mo* months, *NMI* Neuromuscular impairement, *RSV* Respiratory syncytial virus, *SARI* Severe acute respiratory infection, *URTI* Upper respiratory tract infection, *yrs* years

^a^As attack rate (%) or incidence rate (per 100, 100,000 children or whole population)

^b^PCR-tested for RSV, Influenza A/B and SARS-CoV-2

^c^PCR-tested for RSV, Rhino/enterovirus, Influenza A/B, Adenovirus, Norovirus, Rotavirus

^d^PCR-tested for RSV, hMPV, Rhinovirus and other (unspecified) respiratory pathogens

### Results of included studies by outcome measure

#### RSV detection rate by age and setting

RSV detection rates were reported in 33 articles, ranging from 0.1 to 55.4% across heterogenous study populations (detailed description in Supplementary Table S3) [17, 33, 34, 36–39, 41–43, 45, 46, 49–52, 55–59, 61–71]. Within the pediatric population not immunized with palivizumab, RSV detection rates in ARI patients (including case definitions such as acute respiratory tract infection (ARTI), lower respiratory tract infection (LRTI), respiratory symptoms or illness) ranged from 5.2 to 55.4% in inpatient cases [33, 34, 41–43, 45, 56, 57, 59, 61, 65–68, 70, 71] and from 17.6 to 20.9% in outpatient cases [41, 42, 59]. Based on data from 2017 to 2022, separate RSV detection rates for children and adults were reported, with rates across respiratory seasons ranging from 25.6 to 48.9% for children and from 3.8 to 9.4% for the adult population [63].

A nationwide surveillance analysis for ALRI described a proportion of RSV among all hospitalized ARI cases of 0.5% as well as 0.1% RSV among all outpatient ARI cases across all age groups [38]. Among these hospitalized RSV cases and outpatient RSV cases, 93% and 66% were younger than 2 years old, respectively.

Considering only studies that reported separate RSV detection rates in hospitals and in the outpatient treatment setting, a range of 9.5–38% for inpatient cases and 17.6–20.9% for outpatient cases was observed [41, 42, 59].

#### RSV incidence

Seven studies reported the incidence of RSV infection, although the reporting format (as attack rates in percent, or rate per 100 or 100,000 children or whole population), case definitions and underlying populations varied widely [36, 42, 45, 50–52, 56]. A surveillance study reported attack rates of RSV-attributable medically attended ARI cases across all age groups with a range of 0.3–1.2% for the seasons 2010–2011 to 2017–2018. Looking at age-specific attack rates across seasons, higher rates were reported for the age groups 0–1 years (range: 4.0-14.8%) and 2–4 years (range: 4.0‐10.3%) compared with older age groups (range of > 60 years: 0.1–1.2%) [36].

A further study calculated the incidence of RSV-related LRTI outpatient consultations and hospitalizations in children aged ≤ 3 years from 1999 to 2001 and reported 7.7 outpatient consultations per 100 children and 1,117 hospitalizations per 100,000 children. By extrapolating to the entire German population, annual 183,761 outpatient and 26,524 hospitalized cases related to RSV were estimated and reported [42].

All four articles reporting RSV-related hospitalizations among infants and young children (< 25 months of age) with underlying conditions and immunized with palivizumab, were based on the “German Synagis Registry”. For this restricted population, the reported RSV-related hospitalization rates ranged from 0.7 to 1.6%. [49–52].

#### RSV seasonality

A total of 18 reports on seasonality were identified [17, 33, 34, 37, 39, 43, 44, 46, 54, 55, 57, 62–64, 67–70], presenting different stratification, such as per calendar year or per season, per month or per.

**Fig. 2:**
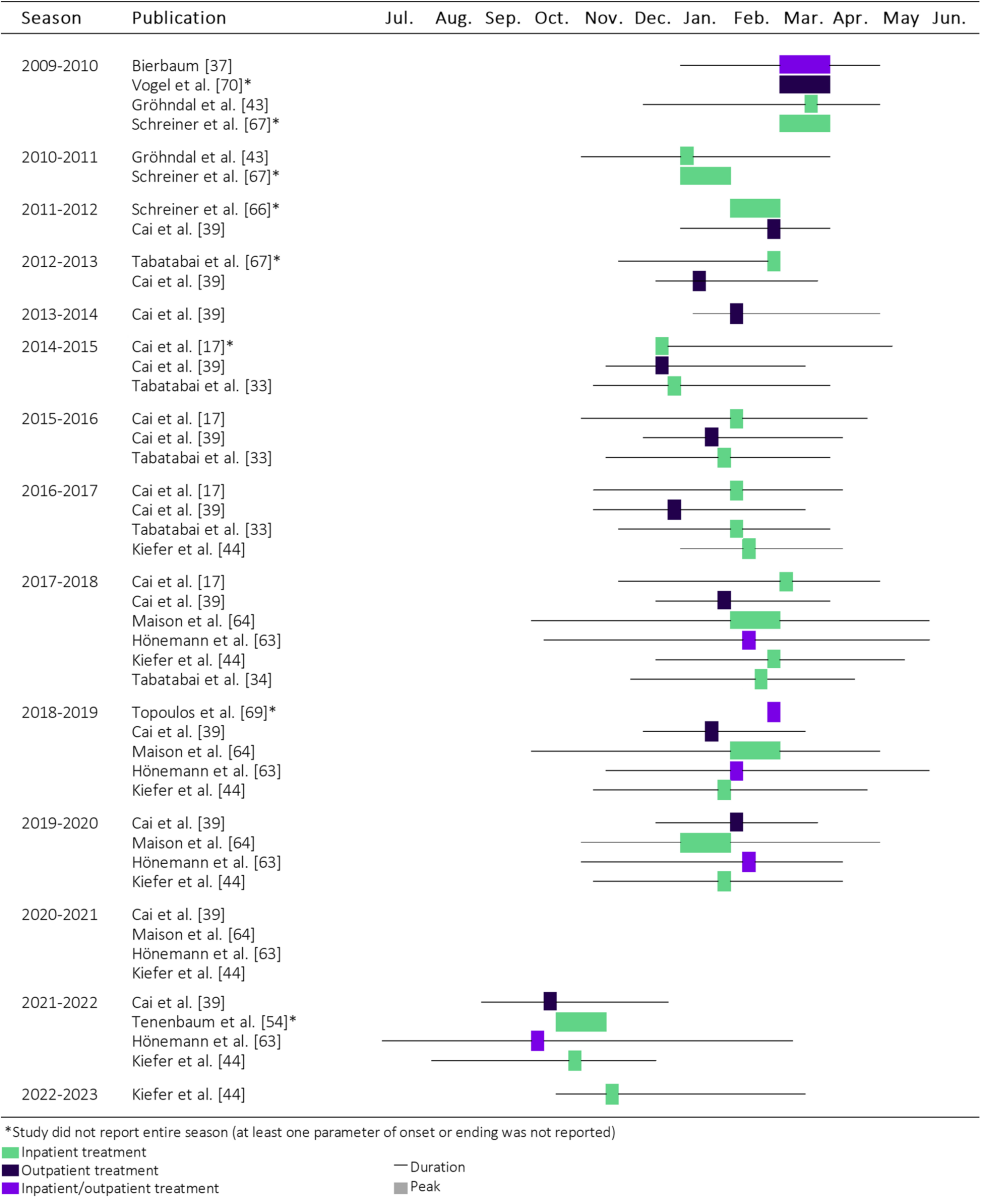
Seasonal RSV activity reported in included articles by publication and year (based on month or calendar weeks), Germany 2009–2023. RSV activity reported for seasons prior to 2009 were not depicted

calendar week. Figure 2 shows RSV activity onset, peak and ending for each season from 2003 to 2023. In the pre-COVID-19 pandemic seasons, the RSV epidemic seasons were most frequently reported to start in November, to peak in February and to end in April. During the COVID-19 pandemic, the typical seasonality was disrupted, as no noticeable season during the winter of 2020–2021 and an early peak of season 2021–2022 was reported.

#### Clinical manifestation of RSV infection

Clinical manifestations of RSV-typical presentations, including pneumonia, bronchitis, and bronchiolitis were reported in 19 articles [19, 32–34, 38, 41, 42, 45, 49–51, 53, 61–63, 68–71]. Among these 19 articles, the proportion of RSV cases with pneumonia varied widely, ranging from 6.9 to 34.2% in pediatric outpatient cases, from to 12.2–61.3% in pediatric inpatient cases, and from 32.5 to 50% in adult inpatient cases. No study reported on the proportion of RSV cases with pneumonia in adult outpatient cases separated from pediatric cases. A single-center study provided comparative data between the adult and pediatric population with RSV in the inpatient setting, revealing that 34.2% of adults (≥ 18 years) had pneumonia, whereas the proportion among children < 18 years of age was 19.0% [63]. Another single-center study investigating proportions of pneumonia within the pediatric population with RSV indicated that 19.4% of infants < 6 months of age opposed to 50% of children aged 2–5 years had pneumonia [19].

Bronchiolitis proportions among RSV cases were documented in 9 articles, showing a range of 18–62.8% across all ages [19, 33, 38, 41, 42, 50, 51, 61, 71]. When stratifying by age groups within a pediatric population from a single-center study, infants had higher proportions of bronchiolitis (79.6% and 81.3% of children aged < 6 months and 6-<12 months, respectively) than young children (51.3% and 39.7% of children aged 1-<2 years and 2–5 years, respectively) [19].

Bronchitis was reported in 10 articles, with its occurrence ranging from 4.5 to 37% among RSV-related cases [19, 32, 33, 38, 41, 42, 50, 51, 61, 63, 71]. Additionally, wheezing bronchitis was classified in 1 article, occurring in 33.6% of RSV-positive hospitalized children < 2 years of age.

Five studies reported the combined prevalence of RSV-related bronchitis and bronchiolitis in children [34, 45, 53, 63, 68], ranging from 41.7 to 80.8%. Finally, four further studies reported cases of laryngotracheobronchitis, commonly known as croup, with proportions between 0.0 and 13.8% in pediatric inpatient and 12.0–14.1% in pediatric outpatient cases [32, 41, 42, 71].

#### Underlying conditions

Thirteen articles reported underlying conditions of individuals with RSV infection, mostly focusing on the pediatric RSV population in the inpatient treatment setting (Supplementary Table S3) [17, 19, 35, 44, 45, 47–50, 52, 53, 60, 63].

Within the pediatric RSV population (excluding the palivizumab-eligible populations [49, 50, 52]), premature birth (including varying definitions of birth before 35–37 weeks of gestation) was the most frequently reported underlying condition with a wide range of proportions from 4.2 to 41.1% due to heterogenous populations [19, 35, 44, 48, 53, 60, 63]. The higher values for the proportion of prematurity in RSV cases were due to subpopulations of patients with further existing underlying conditions. In contrast, in articles considering study populations without specific further underlying conditions other than prematurity, pre-term status ranged from 12.7 to 24.1% among hospitalized RSV cases [19, 35, 44, 48, 53, 63], was 25.0% among ICU-admitted [53], and 4.2% in outpatient RSV cases [53]. Like prematurity, proportions of RSV cases with CHD (range: 1.6–26.0% [17, 19, 44, 47, 48, 60]) and chronic lung disease (CLD)/BPD (range: 1.4–21.6% [19, 35, 45, 48, 60]) varied widely due to heterogenous populations, and partially had coexisting underlying conditions. In three included articles, all based on data from the “German Synagis Registry”, analyzing RSV-related hospitalizations of children immunized with palivizumab, prematurity was the most frequently occurring underlying condition (78.8% and 88% [49, 50, 52]), followed by CLD/BPD (41.2% and 47% [49, 50]) and CHD (range: 26.2–34.2% [49, 50, 52]).

Within the adult RSV population, the highest reported proportions of underlying conditions were cardiovascular disease (53.6% and 58.8% [44, 63]), diabetes (30.4% and 31.2% [44, 63]), COPD (17.1% and 28.7% [44, 63]) and immunosuppression (38.0% [63]).

#### Mortality

Eight articles reported data on mortality in the studied population with RSV infection, with case-fatality rates ranging from 0.0 to 0.4% among hospitalized children with RSV [17, 19, 47, 48, 60, 62, 63, 69]. Among children with underlying conditions, hospitalized RSV cases were fatal in 1.2% of preterm cases [47] and 5.5% of children with neuromuscular impairments (NMI) [60]. However, logistic regression analyses indicated that NMI was indirectly associated with RSV-related death due to increased risk of PICU admission and respiratory failure [60]. The reported lethality of RSV infection in adult populations was higher than in children, with in-hospital case-fatality rates ranging from 6.1% (in inpatient cases with RSV and a mean age of > 60 years) to 13.3% (RSV-positive pneumonia inpatient cases) [63, 69].

One study reported age distribution and underlying conditions in deceased RSV cases. 40% of deceased RSV cases were below 4 years of age, while 12% were 5–64 years and 48% above 65 years of age. 60% of deceased RSV cases had an underlying cardiovascular disease [17].

#### RSV-related health care resource use

**Fig. 3:**
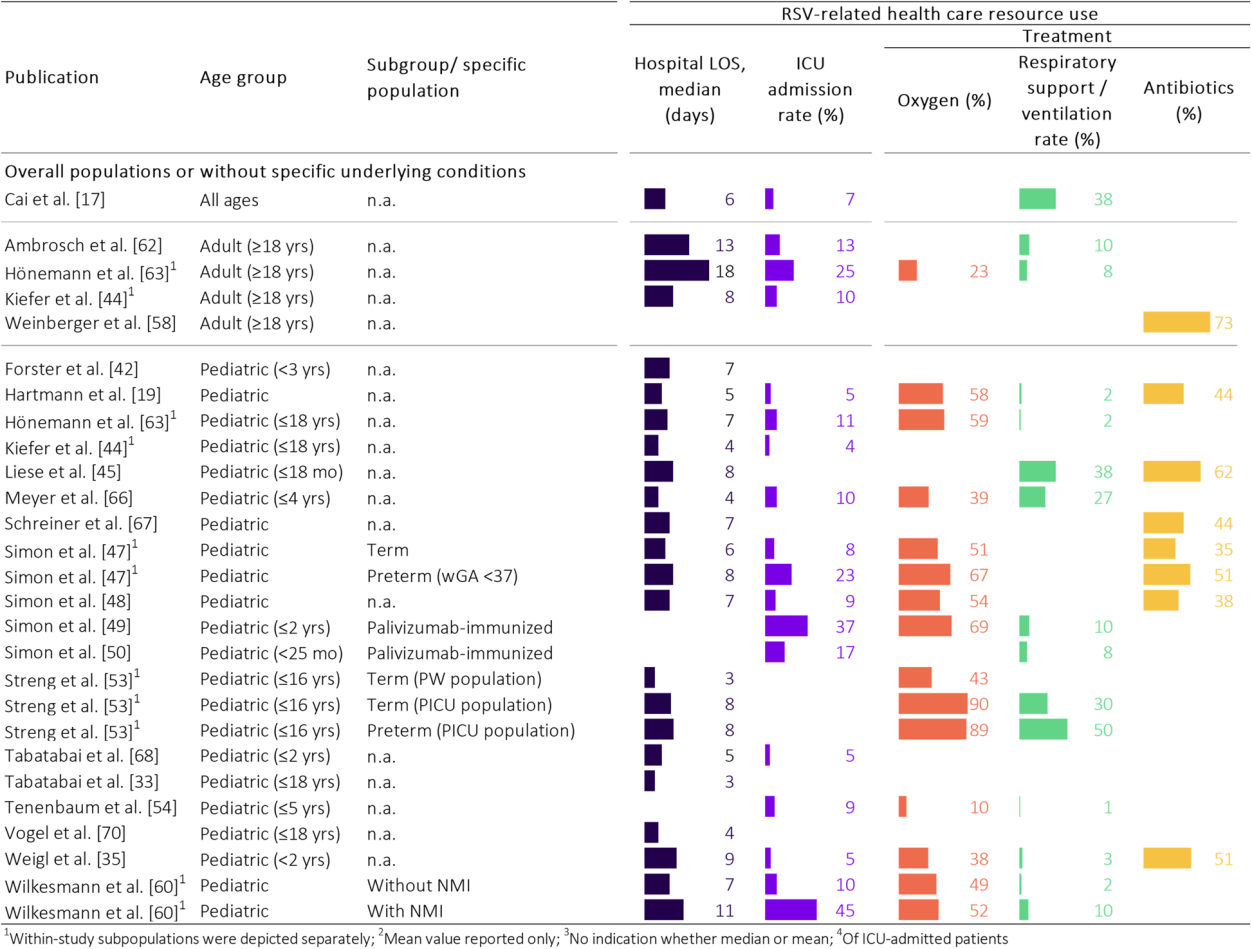
Overview of health care resource use (HCRU) reported in included articles by publication, age group and study population. LOS: length of stay; NMI: neuromuscular impairment; RSV: respiratory syncytial virus; (P)ICU: (pediatric) intensive care unit; PW: pediatric hospital ward; wGA: gestational age in weeks; yrs: years

Twenty-three articles reported health care resource use (HCRU) associated with RSV infection meeting our pre-defined inclusion criteria, such as ICU admission rates, length of stay (LOS) in the hospital or at the ICU, and treatment options (inhalation, oxygen, ventilation, respiratory support, antibiotics, duration of therapy; Fig. 3) [17, 19, 33, 35, 40, 42, 44, 45, 47–50, 53, 54, 58, 60, 62, 63, 65–68, 70].

Hospital LOS of RSV cases was reported in 16 articles [17, 19, 33, 35, 42, 44, 45, 47, 48, 53, 60, 62, 63, 66–68, 70]. The median LOS ranged from 3 to 11 days, while the mean LOS ranged from 5 to 18.3 days across all populations. Two articles have reported the hospital LOS for both children and adults within the same study, in which the hospital LOS was higher in adults (mean 18.3 days, median 8 days) than in children (mean 6.5 days, median 4 days) [44, 63].

ICU admission rates of hospitalized RSV cases were reported in 14 articles, varying from 3.6 to 45% [17, 19, 35, 44, 47–50, 54, 60, 62, 63, 66, 68]. Within pediatric populations, subgroups of children with underlying conditions had the highest ICU admission rates with up to 45% (Fig. 3). In children without underlying conditions, the youngest age groups were reported to be admitted most frequently to the ICU (9.3% of children < 6 months of age [19]). Comparing the entire pediatric (< 18 years) and adult (≥ 18 years) population within the same respective studies, adults were admitted to the ICU more frequently than children (25.0% and 10.1% for adults vs. 10.5% and 3.6% for children) [44, 63]. The reported ICU LOS was consistent with 8.8 to 9 days (mean) or 4.5 to 5 days (median) in children, and 7.6 days (mean) in adults [17, 53, 63].

In the included articles, the reported treatment covered options such as oxygen supply, (non-invasive) respiratory support and (mechanical) ventilation, including high-flow nasal cannula (HFNC), continuous positive airway pressure (CPAP), intermittent mandatory ventilation (IMV), inhalation, and administration of antibiotics. Ventilation among children hospitalized with RSV was applied in 1.3–9.6% of cases, while two studies reported ventilation rates of 10.6% and 38% in ICU-admitted children with RSV [17, 53]. The term “ventilation” was specified differently in the various articles, with some articles referring to both non-invasive and mechanical, i.e. invasive ventilation, separately. Other articles did not further specify the term “ventilation”. Three articles reported specific methods, such as HFNC (3.5% and 24.3% of hospitalized RSV cases) and CPAP (1.8% of RSV cases and 34.6% of ICU-admitted cases with RSV-A) [19, 53, 66]. In addition to respiratory-related treatment options, the use of antibiotics was reported in 7 articles, being administered to 35–62.2% of pediatric RSV hospitalized and 73% of adult RSV outpatient cases [19, 35, 45, 47, 48, 58, 67].

Two articles provided detailed information on primary care visits, reporting a mean of 2.7 (< 3 years) and 4.3 (≥ 18 years) additional consultations for RSV outpatient cases after initial presentation to the general practitioner, and a mean of 3.6 consultations for RSV inpatient cases after discharge [42, 58].

#### RSV-related costs

Our search identified only 1 article reporting costs associated with RSV-LRTI in children ≤ 3 years of age from 1999 to 2001, estimating mean costs of €163 per outpatient case and €2.772 per hospitalized RSV case, including direct and indirect costs. Extrapolating costs per case to the entire German population, annual costs of €17.5 million associated with outpatient and €66 million for inpatient treatment of RSV cases were reported [40].

## Discussion

This is the first systematic literature review synthesizing the evidence of RSV-associated burden of disease for Germany and describing the heterogeneity in study methods, settings, and populations, as well as reporting a wide range of outcome measures. Most studies were conducted in pediatric populations and in the inpatient setting. The RSV detection rate was the most frequently reported outcome measure followed by information about HCRU associated with RSV infection, both for diverse study populations.

Outcome measures such as the incidence of RSV infection, the number of hospitalizations, or the ventilation rate, were reported to be highest in infants and young children. Severe RSV cases were reported for both infants and young children as well as in the elderly. However, RSV-related hospitalization in older adults were reported to have higher case-fatality rates and ICU admission rates than in children. This variation of outcomes by age in included studies is in line with European multinational studies describing the RSV disease burden among children [7, 8, 72], adults [73] or both children and adults [74].

In the included studies, premature birth, CHD, and CLD in children, as well as chronic diseases like cardiovascular issues and immunosuppression in older adults, were reported to increase the individual risk of severe RSV-related complications. This is in line with existing evidence of underlying conditions as risk factors for severe RSV disease in infants and young children [75–78]. RSV has also been found to exacerbate existing chronic respiratory disease and asthma in adults, leading to increased morbidity and mortality [15]. Although premature infants or children with underlying conditions are particularly affected by severe RSV infection, several studies have shown that most infants contracting RSV do not have any underlying conditions [35, 44]. This highlights that RSV is not confined to children with specific underlying conditions but can equally affect otherwise healthy children, which is supported by international and recently published literature from Germany. An analysis published in late 2023 based on the Hospital Statistics Database of the German Federal Statistical Office showed that in Germany most RSV cases between 2010 and 2019 occurred in children < 4 years of age that were otherwise healthy [79]. A retrospective analysis of RSV-associated hospitalizations data from the French Hospital database found that 87% of RSV-associated hospitalized children were otherwise healthy and that almost half were born out of the RSV season [5]. Analyses for otherwise healthy children and infants born out of season are missing for Germany.

With 7 out of 42 articles, only few calculated the incidence of RSV-specific outcomes such as infection or hospitalizations and extrapolated the numbers to Germany. Only 1 article estimated annual incidence of RSV-related ALRI hospitalization and outpatient cases for children aged 3 years and younger [42]. In another article, annual attack rates of RSV-attributable medically attended ARI cases were calculated for all age groups [36], which were derived from outpatient data from the German influenza sentinel system of the Robert Koch Institute. The surveillance system also monitors excess consultations caused by RSV infection, which may be another useful parameter to quantify the outpatient burden [28]. Understanding the population level incidence of RSV is critical to estimate the RSV burden on health care systems and possible impact of future immunizations.

We identified only few studies using routine data, and no health insurance claims data analysis. The conduct of claims data analyses or cohort studies with a follow-up period of several years could complement existing evidence to identify and quantify long-term sequelae following a RSV infection, like recurrent wheezing or asthma, which is discussed as a consequence after early-life LRTI-RSV infection [80, 81], but not reported in any of the included studies. Two ICD-10 code-based studies were included. For those types of studies data validity of the used codes should be considered. One of the included studies from 2020 evaluated retrospectively the use of RSV-specific ICD-10 codes for an existing RSV surveillance system in Germany [38]. The authors found that RSV-specific ICD-10 codes (J12.1, J20.5 and J21.0) had poor sensitivity of 6% and high specificity of 99.8%. The combination of RSV-specific and selected non-RSV-specific ALRI ICD-10 codes, though, improved sensitivity to 44% while maintaining high specificity (91%).

Few studies reported on RSV-attributable mortality. In children, case-fatality was reported to be very low, except for subgroups with severe underlying conditions (up to 5.5% in children with NMI [60]), as it is in line with other high-income countries [1, 3]. Globally, the highest burden of RSV-related mortality in children occurs in low- and middle-income countries [1]. In the included studies, the adult population had the highest case-fatality rates (11.3–13.3%), which were often high in those groups with high proportions of RSV-pneumonia [62, 69]. The only included study that described the RSV disease burden compared with the disease burden of influenza in elderly aged ≥ 60 years was the study by an der Heiden et al., which covered the 2010–2011 to 2017–2018 seasons [36]. The authors found higher influenza attack rates than RSV attack rate. This is in line with a study from 2023 conducted in the US that found that among hospitalized older adults, RSV was less common than with COVID-19 or influenza. However, they found higher disease severity in older adults hospitalized with RSV than with COVID-19 or influenza [82].

Many articles reported on seasonality but had different definitions for the onset and ending of RSV activity or depicted seasonality in various ways. This may have translated into inconsistent seasonal patterns within the same years across different articles. A homogenized definition and presentation as proposed in Cai et al. (2022) [39] may facilitate the detection of seasonal patterns (severe vs. less severe, early vs. late onset) and thus supporting strategies for health care and prevention to mitigate the disease burden in the future.

There is limited evidence on the economic burden of RSV in Germany, with only one outdated study included in this review [40]. However, in late 2023, two exhaustive studies were published, one that analyzed RSV-related hospitalization costs in all age groups for 2010–2019, the other in children < 2 years of age for 2019–2022 [79, 83]. From a third-party payer perspective, the mean costs of RSV hospitalization ranged between €3,443 in 1–4-year-olds and €7,339 among adults during 2010–2019, and between €3,001 and €3,961 in children ≤ 2 years during 2019–2022 [79, 83]. With recent new interventions to prevent severe RSV infection in infants, young children, and the elderly, reevaluated costs should be considered, as health economics plays a key role in health care decision-making.

This review’s primary strength is its broad inclusion criteria. The synthesized results come from a variety of populations (infants, children, adults, elderly, patients with conditions), treatment settings (in- and outpatient cases), event types (any ALRI, influenza-like illness) and regions (distributed all over Germany). Most of the included studies had large populations of more than 300 patients, 12 studies were nationwide, allowing this systematic literature review to provide a robust description of the epidemiological burden of RSV disease. In addition to the comprehensive collection of evidence, the extensiveness of this systematic review enabled the detection of evidence gaps. Data on RSV in children without underlying diseases, RSV in the elderly, on the economic burden caused by RSV infection, on RSV burden in the outpatient setting, and on tracking long-term sequalae after RSV infection were scarce to unavailable.

Due to high variability and heterogenous nature of the research question, this review is limited by the inability to assess quality of the included studies nor to conduct a meta-analysis. We found heterogeneity in reported outcome measures and study populations across included studies, such as the included type of disease within the population, different classification of underlying conditions (different definitions or not specified definitions for prematurity), different stratifications of age groups and undefined classification of treatment (e.g. ventilation, being invasive or non-invasive). Second, taking the vast heterogeneity of study designs and their divergent focus into account, we should be cautious in our interpretations of results, especially when summarizing or comparing results across different studies. Finally, this review only considers scholarly articles reflected in PubMed and Embase and does not include other databases and grey literature.

### Future work

The compilation of all available data from included studies showed some similarities, such as the HCRU of individuals with RSV infection within similar populations, but mostly high variability occurred when similar outcome measures were summarized across studies and populations. This strengthens the need to report distinct values for specified subpopulations, which can be stratified by age, treatment setting, underlying conditions, or clinical manifestation to help to quantify the true burden of RSV in Germany and thus health care decision-making for each population. This requires also further studies that quantify the direct and indirect costs associated with RSV.

## Conclusion

Our results demonstrate that RSV causes substantial morbidity in affected individuals in Germany, primarily in infants, young children, and in the elderly, indicating that RSV prevention could have a high impact on individual and public health. Most articles reporting on pediatric RSV cases, focused on children with specific underlying conditions. Those few also focusing on children without any underlying conditions reported high proportions of RSV cases in children. Until now, only few studies described the burden of RSV infection in otherwise healthy children or the elderly. Considering the remarkably high reported burden, these groups should be further investigated in future studies.

### Supplementary Information

Additional file 1: Supplementary Table S1. Search terms

Additional file 2: Supplementary Table S2. Inclusion and exclusion criteria

Additional file 3: Supplementary Table S3. Extracted characteristics and RSV-related outcomes of 42 included studies in detail

## Data Availability

No datasets were generated or analysed during the current study.

## Abbreviations

ALRI: Acute lower respiratory tract infection
ARI: Acute respiratory
BPD: Bronchopulmonary disease
CHD: Congenital heart disease
CLD: Chronic lung disease
CPAP: Continuous positive airway pressure
HCRU: Health care resource use
HFNC: High-flow nasal cannula
ICU: Intensive care unit
IMV: Intermittent mandatory ventilation
LOS: Length of stay
LRTI: Infection lower respiratory tract infection
NMI: Neuromuscular impairment
PRISMA: Preferred Reporting Items for Systematic reviews and Meta-Analyses
RSV: Respiratory syncytial virus

## References

[1] Shi T, McAllister DA, O’Brien KL, Simoes EAF, Madhi SA, Gessner BD, et al. Global, regional, and national disease burden estimates of acute lower respiratory infections due to respiratory syncytial virus in young children in 2015: a systematic review and modelling study. Lancet. 2017;390(10098):946–58. 10.1016/S0140-6736(17)30938-8. 28689664 10.1016/S0140-6736(17)30938-8 PMC5592248

[2] Staadegaard L, Caini S, Wangchuk S, Thapa B, de Almeida WAF, de Carvalho FC, et al. Defining the seasonality of respiratory syncytial virus around the world: National and subnational surveillance data from 12 countries. Influenza Other Respir Viruses. 2021;15(6):732–41. 10.1111/irv.12885. 34255934 10.1111/irv.12885 PMC8542954

[3] Li Y, Wang X, Blau DM, Caballero MT, Feikin DR, Gill CJ, et al. Global, regional, and national disease burden estimates of acute lower respiratory infections due to respiratory syncytial virus in children younger than 5 years in 2019: a systematic analysis. Lancet. 2022;399(10340):2047–64. 10.1016/S0140-6736(22)00478-0. 35598608 10.1016/S0140-6736(22)00478-0 PMC7613574

[4] Obando-Pacheco P, Justicia-Grande AJ, Rivero-Calle I, Rodriguez-Tenreiro C, Sly P, Ramilo O, et al. Respiratory Syncytial Virus Seasonality: A Global Overview. J Infect Dis. 2018;217(9):1356–64. 10.1093/infdis/jiy056. 29390105 10.1093/infdis/jiy056

[5] Demont C, Petrica N, Bardoulat I, Duret S, Watier L, Chosidow A, et al. Economic and disease burden of RSV-associated hospitalizations in young children in France, from 2010 through 2018. BMC Infect Dis. 2021;21(1):730. 10.1186/s12879-021-06399-8. 34340679 10.1186/s12879-021-06399-8 PMC8327424

[6] Duan Y, Jiang M, Huang Q, Jia M, Yang W, Feng L. Incidence, hospitalization, and mortality in children aged 5 years and younger with respiratory syncytial virus-related diseases: a systematic review and meta-analysis. Influenza Other Respir Viruses. 2023;17(5):e13145. 10.1111/irv.13145. 37223668 10.1111/irv.13145 PMC10201211

[7] Del Riccio M, Spreeuwenberg P, Osei-Yeboah R, Johannesen CK, Vazquez Fernandez L, Teirlinck AC, et al. Defining the Burden of Disease of RSV in the European Union: estimates of RSV-associated hospitalisations in children under 5 years of age. A systematic review and modelling study. J Infect Dis. 2023. 10.1093/infdis/jiad188. 37246724 10.1093/infdis/jiad188 PMC10681872

[8] Bont L, Checchia PA, Fauroux B, Figueras-Aloy J, Manzoni P, Paes B, et al. Defining the epidemiology and burden of severe respiratory syncytial virus infection among infants and children in Western Countries. Infect Dis Ther. 2016;5(3):271–98. 10.1007/s40121-016-0123-0. 27480325 10.1007/s40121-016-0123-0 PMC5019979

[9] Glezen WP, Taber LH, Frank AL, Kasel JA. Risk of primary infection and reinfection with respiratory syncytial virus. Am J Dis Child. 1986;140(6):543–6. 10.1001/archpedi.1986.02140200053026. 3706232 10.1001/archpedi.1986.02140200053026

[10] Meissner HC. Viral bronchiolitis in children. N Engl J Med. 2016;374(1):62–72. 10.1056/NEJMra1413456. 26735994 10.1056/NEJMra1413456

[11] Shi T, Denouel A, Tietjen AK, Campbell I, Moran E, Li X, et al. Global Disease Burden estimates of respiratory Syncytial Virus-Associated Acute respiratory infection in older adults in 2015: a systematic review and Meta-analysis. J Infect Dis. 2020;222(Suppl 7):S577–83. 10.1093/infdis/jiz059. 30880339 10.1093/infdis/jiz059

[12] Baraldi E, Bonadies L, Manzoni P. Evidence on the link between respiratory syncytial virus infection in Early Life and Chronic Obstructive Lung diseases. Am J Perinatol. 2020;37(S 02):S26–30. 10.1055/s-0040-1714345. 32772357 10.1055/s-0040-1714345

[13] Sigurs N, Gustafsson PM, Bjarnason R, Lundberg F, Schmidt S, Sigurbergsson F, et al. Severe respiratory syncytial virus bronchiolitis in infancy and asthma and allergy at age 13. Am J Respir Crit Care Med. 2005;171(2):137–41. 10.1164/rccm.200406-730OC. 15516534 10.1164/rccm.200406-730OC

[14] CDC. RSV in Older Adults and Adults with Chronic Medical Conditions. https://www.cdc.gov/rsv/high-risk/older-adults.html Accessed 26 October 2023.

[15] Ackerson B, Tseng HF, Sy LS, Solano Z, Slezak J, Luo Y, et al. Severe morbidity and mortality Associated with respiratory Syncytial Virus Versus Influenza infection in hospitalized older adults. Clin Infect Dis. 2019;69(2):197–203. 10.1093/cid/ciy991. 30452608 10.1093/cid/ciy991 PMC6603263

[16] Krilov LR, Roberts NJ. Jr. Respiratory syncytial virus (RSV) update. Viruses. 2022;14(10). 10.3390/v14102110. 10.3390/v14102110 PMC9607319 36298665

[17] Cai W, Buda S, Schuler E, Hirve S, Zhang W, Haas W. Risk factors for hospitalized respiratory syncytial virus disease and its severe outcomes. Influenza Other Respir Viruses. 2020b;14(6):658–70. 10.1111/irv.12729. 32064773 10.1111/irv.12729 PMC7578333

[18] Shmueli E, Goldberg O, Mei-Zahav M, Stafler P, Bar-On O, Levine H, et al. Risk factors for respiratory syncytial virus bronchiolitis hospitalizations in children with chronic diseases. Pediatr Pulmonol. 2021;56(7):2204–11. 10.1002/ppul.25435. 33913611 10.1002/ppul.25435

[19] Hartmann K, Liese JG, Kemmling D, Prifert C, Weissbrich B, Thilakarathne P, et al. Clinical burden of respiratory Syncytial Virus in Hospitalized Children aged =5 years (INSPIRE study)</at. J Infect Dis. 2022;226(3):386–95. 10.1093/infdis/jiac137. 35417015 10.1093/infdis/jiac137 PMC9417125

[20] Arriola CS, Kim L, Langley G, Anderson EJ, Openo K, Martin AM, et al. Estimated Burden of Community-Onset Respiratory Syncytial Virus-Associated hospitalizations among children aged < 2 years in the United States, 2014-15. J Pediatr Infect Dis Soc. 2020;9(5):587–95. 10.1093/jpids/piz087. 10.1093/jpids/piz087 PMC7107566 31868913

[21] Rha B, Curns AT, Lively JY, Campbell AP, Englund JA, Boom JA, et al. Respiratory Syncytial Virus-Associated hospitalizations among Young children: 2015–2016. Pediatrics. 2020;146(1). 10.1542/peds.2019-3611. 10.1542/peds.2019-3611 PMC12874392 32546583

[22] Meyer KC. The role of immunity in susceptibility to respiratory infection in the aging lung. Respir Physiol. 2001;128(1):23–31. 10.1016/s0034-5687(01)00261-4. 11535259 10.1016/s0034-5687(01)00261-4 PMC7130717

[23] Falsey AR, Hennessey PA, Formica MA, Cox C, Walsh EE. Respiratory syncytial virus infection in elderly and high-risk adults. N Engl J Med. 2005;352(17):1749–59. 10.1056/NEJMoa043951. 15858184 10.1056/NEJMoa043951

[24] Messina A, Germano C, Avellis V, Tavella E, Dodaro V, Massaro A, et al. New strategies for the prevention of respiratory syncytial virus (RSV). Early Hum Dev. 2022;174:105666. 10.1016/j.earlhumdev.2022.105666. 36174288 10.1016/j.earlhumdev.2022.105666

[25] Mazur NI, Terstappen J, Baral R, Bardaji A, Beutels P, Buchholz UJ, et al. Respiratory syncytial virus prevention within reach: the vaccine and monoclonal antibody landscape. Lancet Infect Dis. 2023;23(1):e2–21. 10.1016/S1473-3099(22)00291-2. 35952703 10.1016/S1473-3099(22)00291-2 PMC9896921

[26] European Medicines Agency. Synagis - palivizumab. Annex I - Summary of product characteristics. https://www.ema.europa.eu/en/documents/product-information/synagis-epar-product-information_en.pdf. Accessed 22 July 2024.

[27] Gemeinsamer Bundesausschuss. Bekanntmachung eines Beschlusses des Gemeinsamen Bundesausschusses über eine Änderung der Arzneimittel-Richtlinie in Anlage 4: Therapiehinweis zu Palivizumab [BAnz. Nr. 181 (S. 4 260) vom 27.11.2008]. https://www.g-ba.de/downloads/39-261-694/2008-06-19-AMR4-Palivizumab_BAnz.pdf. Accessed 22 July 2024.

[28] Buda S, Preuß U. Robert Koch-Institut. Bericht zur Epidemiologie der Influenza in Deutschland, Saison 2018/19, Berlin. 2019. 10.25646/6232

[29] Robert Koch-Institut. FAQ-Liste zu RSV-Infektionen. https://www.rki.de/SharedDocs/FAQ/RSV/FAQ_Liste_RSV.html. Accessed 26 October 2023.

[30] Moher D, Liberati A, Tetzlaff J, Altman DG, Group P. Preferred reporting items for systematic reviews and meta-analyses: the PRISMA statement. BMJ. 2009;339:b2535. 10.1136/bmj.b2535. 19622551 10.1136/bmj.b2535 PMC2714657

[31] Page MJ, McKenzie JE, Bossuyt PM, Boutron I, Hoffmann TC, Mulrow CD, et al. The PRISMA 2020 statement: an updated guideline for reporting systematic reviews. PLoS Med. 2021;18(3):e1003583. 10.1371/journal.pmed.1003583. 33780438 10.1371/journal.pmed.1003583 PMC8007028

[32] Weigl JA, Puppe W, Schmitt HJ. Can respiratory syncytial virus etiology be diagnosed clinically? A hospital-based case-control study in children under two years of age. Eur J Epidemiol. 2003;18(5):431–9. 10.1023/a:1024213400297. 12889690 10.1023/a:1024213400297

[33] Tabatabai J, Ihling CM, Rehbein RM, Schnee SV, Hoos J, Pfeil J, et al. Molecular epidemiology of respiratory syncytial virus in hospitalised children in Heidelberg, Southern Germany, 2014–2017. Infect Genet Evol. 2022;98:105209. 10.1016/j.meegid.2022.105209. 35032683 10.1016/j.meegid.2022.105209

[34] Tabatabai J, Ihling CM, Manuel B, Rehbein RM, Schnee SV, Hoos J, et al. Viral etiology and clinical characteristics of Acute Respiratory Tract infections in Hospitalized Children in Southern Germany (2014–2018). Open Forum Infect Dis. 2023;10(3):ofad110. 10.1093/ofid/ofad110. 36968956 10.1093/ofid/ofad110 PMC10034757

[35] Weigl JA, Puppe W, Schmitt HJ. Variables explaining the duration of hospitalization in children under two years of age admitted with acute airway infections: does respiratory syncytial virus have a direct impact? Klin Padiatr. 2004;216(1):7–15. 10.1055/s-2004-817688. 14747964 10.1055/s-2004-817688

[36] An der Heiden M, Buchholz U, Buda S. Estimation of influenza- and respiratory syncytial virus-attributable medically attended acute respiratory infections in Germany, 2010/11-2017/18. Influenza Other Respir Viruses. 2019;13(5):517–21. 10.1111/irv.12666. 31339223 10.1111/irv.12666 PMC6692544

[37] Bierbaum S, Forster J, Berner R, Rucker G, Rohde G, Neumann-Haefelin D, et al. Detection of respiratory viruses using a multiplex real-time PCR assay in Germany, 2009/10. Arch Virol. 2014;159(4):669–76. 10.1007/s00705-013-1876-3. 24126621 10.1007/s00705-013-1876-3 PMC7086993

[38] Cai W, Tolksdorf K, Hirve S, Schuler E, Zhang W, Haas W, et al. Evaluation of using ICD-10 code data for respiratory syncytial virus surveillance. Influenza Other Respir Viruses. 2020a;14(6):630–7. 10.1111/irv.12665. 31206246 10.1111/irv.12665 PMC7578302

[39] Cai W, Durrwald R, Biere B, Schweiger B, Haas W, Wolff T, et al. Determination of respiratory syncytial virus epidemic seasons by using 95% confidence interval of positivity rates, 2011–2021, Germany. Influenza Other Respir Viruses. 2022;16(5):854–7. 10.1111/irv.12996. 35485999 10.1111/irv.12996 PMC9343324

[40] Ehlken B, Ihorst G, Lippert B, Rohwedder A, Petersen G, Schumacher M, et al. Economic impact of community-acquired and nosocomial lower respiratory tract infections in young children in Germany. Eur J Pediatr. 2005;164(10):607–15. 10.1007/s00431-005-1705-0. 15965766 10.1007/s00431-005-1705-0

[41] Forster J. Influenza in children: the German perspective. Pediatr Infect Dis J. 2003;22(10 Suppl):S215-7. 10.1097/01.inf.0000092190.43140.f7. 14551478 10.1097/01.inf.0000092190.43140.f7

[42] Forster J, Ihorst G, Rieger CH, Stephan V, Frank HD, Gurth H, et al. Prospective population-based study of viral lower respiratory tract infections in children under 3 years of age (the PRI.DE study). Eur J Pediatr. 2004;163(12):709–16. 10.1007/s00431-004-1523-9. 15372233 10.1007/s00431-004-1523-9

[43] Gröndahl B, Ankermann T, von Bismarck P, Rockahr S, Kowalzik F, Gehring S, et al. The 2009 pandemic influenza A(H1N1) coincides with changes in the epidemiology of other viral pathogens causing acute respiratory tract infections in children. Infection. 2014;42(2):303–8. 10.1007/s15010-013-0545-5. 24150959 10.1007/s15010-013-0545-5 PMC7100052

[44] Kiefer A, Pemmerl S, Kabesch M, Ambrosch A. Comparative analysis of RSV-related hospitalisations in children and adults over a 7 year-period before, during and after the COVID-19 pandemic. J Clin Virol. 2023;166:105530. 10.1016/j.jcv.2023.105530. 37481874 10.1016/j.jcv.2023.105530

[45] Liese JG, Grill E, Fischer B, Roeckl-Wiedmann I, Carr D, Belohradsky BH, et al. Incidence and risk factors of respiratory syncytial virus-related hospitalizations in premature infants in Germany. Eur J Pediatr. 2003;162(4):230–6. 10.1007/s00431-002-1105-7. 12647195 10.1007/s00431-002-1105-7

[46] Reiche J, Schweiger B. Genetic variability of group a human respiratory syncytial virus strains circulating in Germany from 1998 to 2007. J Clin Microbiol. 2009;47(6):1800–10. 10.1128/JCM.02286-08. 19386848 10.1128/JCM.02286-08 PMC2691087

[47] Simon A, Ammann RA, Wilkesmann A, Eis-Hubinger AM, Schildgen O, Weimann E, et al. Respiratory syncytial virus infection in 406 hospitalized premature infants: results from a prospective German multicentre database. Eur J Pediatr. 2007;166(12):1273–83. 10.1007/s00431-007-0426-y. 17943313 10.1007/s00431-007-0426-y

[48] Simon A, Muller A, Khurana K, Engelhart S, Exner M, Schildgen O, et al. Nosocomial infection: a risk factor for a complicated course in children with respiratory syncytial virus infection–results from a prospective multicenter German surveillance study. Int J Hyg Environ Health. 2008;211(3–4):241–50. 10.1016/j.ijheh.2007.07.020. 17869579 10.1016/j.ijheh.2007.07.020

[49] Simon A, Nowak H, Sterz R. Use of palivizumab in Germany: data from 2002–2007. Klin Padiatr. 2011;223(5):292–8. 10.1055/s-0030-1270515. 21509705 10.1055/s-0030-1270515

[50] Simon A, Gehrmann S, Wagenpfeil G, Wagenpfeil S. Use of Palivizumab in Germany - Report from the German Synagis Registry 2009–2016. Klin Padiatr. 2018a;230(5):263–9. 10.1055/a-0595-7771. 29660756 10.1055/a-0595-7771

[51] Simon A, Gehrmann S, Wagenpfeil G, Wagenpfeil S. Risk factors and main indications for Palivizumab Prophylaxis in a second season Population: results from the German Synagis Registry 2009–2016. Pediatr Infect Dis J. 2018b;37(10):987–91. 10.1097/INF.0000000000002133. 30020201 10.1097/INF.0000000000002133

[52] Simon A, Gehrmann S, Wagenpfeil G, Wagenpfeil S. Palivizumab use in infants with Down syndrome-report from the German Synagis Registry 2009–2016. Eur J Pediatr. 2018c;177(6):903–11. 10.1007/s00431-018-3142-x. 29651734 10.1007/s00431-018-3142-x

[53] Streng A, Goettler D, Haerlein M, Lehmann L, Ulrich K, Prifert C, et al. Spread and clinical severity of respiratory syncytial virus a genotype ON1 in Germany, 2011–2017. BMC Infect Dis. 2019;19(1):613. 10.1186/s12879-019-4266-y. 31299924 10.1186/s12879-019-4266-y PMC6624929

[54] Tenenbaum T, Doenhardt M, Diffloth N, Berner R, Armann JP. High burden of RSV hospitalizations in Germany 2021–2022. Infection. 2022;50(6):1587–90. 10.1007/s15010-022-01889-6. 35904753 10.1007/s15010-022-01889-6 PMC9334552

[55] Terletskaia-Ladwig E, Enders G, Schalasta G, Enders M. Defining the timing of respiratory syncytial virus (RSV) outbreaks: an epidemiological study. BMC Infect Dis. 2005;5:20. 10.1186/1471-2334-5-20. 15801975 10.1186/1471-2334-5-20 PMC1084247

[56] Weigl JA, Puppe W, Belke O, Neususs J, Bagci F, Schmitt HJ. Population-based incidence of severe pneumonia in children in Kiel. Ger Klin Padiatr. 2005;217(4):211–9. 10.1055/s-2004-822699. 10.1055/s-2004-822699 16032546

[57] Weigl JA, Puppe W, Meyer CU, Berner R, Forster J, Schmitt HJ, et al. Ten years’ experience with year-round active surveillance of up to 19 respiratory pathogens in children. Eur J Pediatr. 2007;166(9):957–66. 10.1007/s00431-007-0496-x. 17569085 10.1007/s00431-007-0496-x PMC7087302

[58] Weinberger R, Riffelmann M, Kennerknecht N, Hulsse C, Littmann M, O’Brien J, et al. Long-lasting cough in an adult German population: incidence, symptoms, and related pathogens. Eur J Clin Microbiol Infect Dis. 2018;37(4):665–72. 10.1007/s10096-017-3158-6. 29302815 10.1007/s10096-017-3158-6 PMC7088169

[59] Wetzke M, Schutz K, Kopp MV, Seidenberg J, Vogelberg C, Ankermann T, et al. Pathogen Spectra in hospitalised and nonhospitalised children with community-acquired pneumonia. ERJ Open Res. 2023;9(2). 10.1183/23120541.00286-2022. 10.1183/23120541.00286-2022 PMC10009707 36923566

[60] Wilkesmann A, Ammann RA, Schildgen O, Eis-Hubinger AM, Muller A, Seidenberg J, et al. Hospitalized children with respiratory syncytial virus infection and neuromuscular impairment face an increased risk of a complicated course. Pediatr Infect Dis J. 2007;26(6):485–91. 10.1097/INF.0b013e31805d01e3. 17529864 10.1097/INF.0b013e31805d01e3

[61] Alchikh M, Conrad T, Hoppe C, Ma X, Broberg E, Penttinen P, et al. Are we missing respiratory viral infections in infants and children? Comparison of a hospital-based quality management system with standard of care. Clin Microbiol Infect. 2019;25(3):e3809–16. 10.1016/j.cmi.2018.05.023. 10.1016/j.cmi.2018.05.023 29906596

[62] Ambrosch A, Luber D, Klawonn F, Kabesch M. Focusing on severe infections with the respiratory syncytial virus (RSV) in adults: risk factors, symptomatology and clinical course compared to influenza A / B and the original SARS-CoV-2 strain. J Clin Virol. 2023;161:105399. 10.1016/j.jcv.2023.105399. 36863135 10.1016/j.jcv.2023.105399 PMC9927795

[63] Hönemann M, Thiem S, Bergs S, Berthold T, Propach C, Siekmeyer M, et al. In-Depth analysis of the re-emergence of respiratory Syncytial Virus at a Tertiary Care Hospital in Germany in the summer of 2021 after the alleviation of non-pharmaceutical interventions due to the SARS-CoV-2 pandemic. Viruses. 2023;15(4). 10.3390/v15040877. 10.3390/v15040877 PMC10144477 37112857

[64] Maison N, Peck A, Illi S, Meyer-Buehn M, von Mutius E, Hubner J, et al. The rising of old foes: impact of lockdown periods on non-SARS-CoV-2 viral respiratory and gastrointestinal infections. Infection. 2022;50(2):519–24. 10.1007/s15010-022-01756-4. 35076891 10.1007/s15010-022-01756-4 PMC8787179

[65] Mentel R, Ilgert U, Wegner U, Zimmerman K, Bruns R, Gurtler L. Molecular and clinical characteristics of respiratory syncytial virus infections in hospitalized children. Med Microbiol Immunol. 2005;194(1–2):67–71. 10.1007/s00430-003-0215-9. 14722763 10.1007/s00430-003-0215-9

[66] Meyer M, Ruebsteck E, Eifinger F, Klein F, Oberthuer A, van Koningsbruggen-Rietschel S, et al. Morbidity of respiratory syncytial virus (RSV) infections: RSV compared with severe Acute Respiratory Syndrome Coronavirus 2 infections in children aged 0–4 years in Cologne, Germany. J Infect Dis. 2022;226(12):2050–3. 10.1093/infdis/jiac052. 35172330 10.1093/infdis/jiac052 PMC8903412

[67] Schreiner D, Groendahl B, Puppe W, Off HNT, Poplawska K, Knuf M, et al. High antibiotic prescription rates in hospitalized children with human metapneumovirus infection in comparison to RSV infection emphasize the value of point-of-care diagnostics. Infection. 2019;47(2):201–7. 10.1007/s15010-018-1194-5. 30132249 10.1007/s15010-018-1194-5 PMC7100084

[68] Tabatabai J, Prifert C, Pfeil J, Grulich-Henn J, Schnitzler P. Novel respiratory syncytial virus (RSV) genotype ON1 predominates in Germany during winter season 2012-13. PLoS ONE. 2014;9(10):e109191. 10.1371/journal.pone.0109191. 25290155 10.1371/journal.pone.0109191 PMC4188618

[69] Topoulos S, Giesa C, Gatermann S, Fussen R, Lemmen S, Ewig S. Analysis of acute respiratory infections due to influenza virus A, B and RSV during an influenza epidemic 2018. Infection. 2019;47(3):425–33. 10.1007/s15010-018-1262-x. 30649684 10.1007/s15010-018-1262-x

[70] Vogel M, Grund S, Pandey S, Mayatepek E, Schroten H, Tenenbaum T, et al. What we have learned from the Influenza A pH1N1 2009/10 pandemic: high clinical impact of human metapneumovirus and respiratory Syncytial Virus in Hospitalized Pediatric patients. Jpn J Infect Dis. 2016;69(1):6–11. 10.7883/yoken.JJID.2014.424. 25971322 10.7883/yoken.JJID.2014.424

[71] Wasem S, Weichert S, Walther S, Weigl JA, Puppe W, Ihorst G, et al. Lower respiratory tract disease in children: constant pathogens - constant management?! Klin Padiatr. 2008;220(5):291–5. 10.1055/s-2007-990301. 18095251 10.1055/s-2007-990301

[72] Reeves RM, van Wijhe M, Tong S, Lehtonen T, Stona L, Teirlinck AC, et al. Respiratory Syncytial Virus-Associated Hospital admissions in children younger than 5 years in 7 European Countries using routinely collected datasets. J Infect Dis. 2020;222(Suppl 7):S599–605. 10.1093/infdis/jiaa360. 32815542 10.1093/infdis/jiaa360

[73] Osei-Yeboah R, Spreeuwenberg P, Del Riccio M, Fischer TK, Egeskov-Cavling AM, Boas H, et al. Estimation of the number of RSV-associated hospitalisations in adults in the European Union. J Infect Dis. 2023. 10.1093/infdis/jiad189. 37246742 10.1093/infdis/jiad189 PMC10681866

[74] Johannesen CK, van Wijhe M, Tong S, Fernandez LV, Heikkinen T, van Boven M, et al. Age-specific estimates of respiratory Syncytial Virus-Associated hospitalizations in 6 European countries: a Time Series Analysis. J Infect Dis. 2022;226(Suppl 1):S29–37. 10.1093/infdis/jiac150. 35748871 10.1093/infdis/jiac150

[75] Figueras-Aloy J, Manzoni P, Paes B, Simoes EA, Bont L, Checchia PA, et al. Defining the risk and Associated Morbidity and Mortality of severe respiratory syncytial virus infection among Preterm infants without chronic lung disease or congenital heart disease. Infect Dis Ther. 2016;5(4):417–52. 10.1007/s40121-016-0130-1. 27628014 10.1007/s40121-016-0130-1 PMC5125133

[76] Hon KL, Leung TF, Cheng WY, Ko NM, Tang WK, Wong WW, et al. Respiratory syncytial virus morbidity, premorbid factors, seasonality, and implications for prophylaxis. J Crit Care. 2012;27(5):464–8. 10.1016/j.jcrc.2011.12.001. 22227087 10.1016/j.jcrc.2011.12.001

[77] Manzoni P, Figueras-Aloy J, Simoes EAF, Checchia PA, Fauroux B, Bont L, et al. Defining the incidence and Associated Morbidity and Mortality of severe respiratory syncytial virus infection among children with chronic diseases. Infect Dis Ther. 2017;6(3):383–411. 10.1007/s40121-017-0160-3. 28653300 10.1007/s40121-017-0160-3 PMC5595774

[78] Papenburg J, Hamelin ME, Ouhoummane N, Carbonneau J, Ouakki M, Raymond F, et al. Comparison of risk factors for human metapneumovirus and respiratory syncytial virus disease severity in young children. J Infect Dis. 2012;206(2):178–89. 10.1093/infdis/jis333. 22551815 10.1093/infdis/jis333 PMC7114627

[79] Niekler P, Goettler D, Liese JG, Streng A. Hospitalizations due to respiratory syncytial virus (RSV) infections in Germany: a nationwide clinical and direct cost data analysis (2010–2019). Infection. 2023. 10.1007/s15010-023-02122-8. 37973718 10.1007/s15010-023-02122-8 PMC11499329

[80] Brown PM, Schneeberger DL, Piedimonte G. Biomarkers of respiratory syncytial virus (RSV) infection: specific neutrophil and cytokine levels provide increased accuracy in predicting disease severity. Paediatr Respir Rev. 2015;16(4):232–40. 10.1016/j.prrv.2015.05.005. 26074450 10.1016/j.prrv.2015.05.005 PMC4656140

[81] Fusco F, Hocking L, Stockwell S, Bonsu M, Marjanovic S, et al. The burden of respiratory syncytial virus: understanding impacts on the NHS, society and economy. Rand Health Q. 2022;10(1):2 PMID: 36484078. 36484078 10.7249/rhq-10-01-02 PMC9718057

[82] Surie D, Yuengling KA, DeCuir J, Zhu Y, Gaglani M, Ginde AA, et al. Disease Severity of Respiratory Syncytial Virus compared with COVID-19 and Influenza among hospitalized adults aged >/=60 years - IVY Network, 20 U.S. States, February 2022-May 2023. MMWR Morb Mortal Wkly Rep. 2023;72(40):1083–8. 10.15585/mmwr.mm7240a2. 37796753 10.15585/mmwr.mm7240a2 PMC10564326

[83] Wick M, Poshtiban A, Kramer R, Bangert M, Lange M, Wetzke M, et al. Inpatient burden of respiratory syncytial virus in children =2 years of age in Germany: a retrospective analysis of nationwide hospitalization data, 2019–2022</at. Influenza Other Respir Viruses. 2023;17(11):e13211. 10.1111/irv.13211. 38019702 10.1111/irv.13211 PMC10667831

