# Supplementary material for: Burden of respiratory syncytial virus (RSV) infection in Germany: a systematic review
Source: BMC Infect Dis. 2024 Aug 20;24:844. doi: 10.1186/s12879-024-09758-3 (PMC11337829; doi:10.1186/s12879-024-09758-3)
Supplement: Supplementary file 1 — Additional file 1: Supplementary Table S1. Search terms [file 12879_2024_9758_MOESM1_ESM.pdf]

Supplementary Table S1 Search terms

	RSV		Germany	
	**PubMed**	**Embase**	**PubMed**	**Embase**
**Controlled terms**<br>MeSH (PubMed)	Respiratory Syncytial Viruses	'Human respiratory syncytial virus'	Germany	'Germany'
Emtree (Embase)	Respiratory Syncytial Virus Infections	'Respiratory syncytial virus infection'		'German'
**Free-text terms** (restricted to titles and abstracts)	"RSV"	'RSV'	"Germany"	'Germany'
	"Respiratory syncytial virus"	'Respiratory syncytial virus'	"German"	'German'
Search in both databases was limited to 1 January 2003 to 31 May 2023				

MeSH, medical subject headings; RSV, respiratory syncytial virus

<u>Final search string:</u>

("respiratory syncytial viruses"[MeSH Terms] OR "respiratory syncytial virus infections"[MeSH Terms] OR "RSV"[Title/Abstract] OR "respiratory syncytial virus"[Title/Abstract])

AND

("Germany"[MeSH Terms] OR ("Germany"[Title/Abstract] OR "German"[Title/Abstract])
