# Supplementary material for: Burden of respiratory syncytial virus (RSV) infection in Germany: a systematic review
Source: BMC Infect Dis. 2024 Aug 20;24:844. doi: 10.1186/s12879-024-09758-3 (PMC11337829; doi:10.1186/s12879-024-09758-3)
Supplement: Supplementary file 2 — Additional file 2: Supplementary Table S2. Inclusion and exclusion criteria [file 12879_2024_9758_MOESM2_ESM.pdf]

Supplementary Table S2 Inclusion and exclusion criteria

Criteria	Inclusion	Exclusion
Country	Germany	All other countries
Population	All ages and populations	None
Pathogen	Respiratory syncytial virus (RSV)	All other pathogens
Outcomes	*Epidemiology*<br>• Incidence<br>• RSV detection/positivity rate (% of acute respiratory tract infection)<br>• Seroprevalence<br>• Age distribution of RSV cases<br>• Distribution of RSV cases across age groups, seasons and other characteristics<br>• Underlying conditions (prematurity, chronic heart diseases, pulmonary disease, diabetes, immunosuppression)<br>• Seasonality	*Exclusive focus of outcomes on:*<br>• Phylogenetic analysis<br>• Duration of viral shedding<br>• Risk factor of RSV infection and predictors of severe disease course<br>• RSV detection/positivity rate[a]
	Clinical manifestation<br>• Bronchiolitis<br>• Bronchitis<br>• Pneumonia<br>• Laryngotracheobronchitis	*Exclusive focus of outcomes on:*<br>• Signs and symptoms without reporting specific diagnoses<br>• Radiological findings
	Resource use and costs<br>• Hospitalization<br>• Intensive care unit admission<br>• Length of stay<br>• Treatment (inhalation, oxygen therapy, ventilation, antibiotics)<br>• Costs	
Study design	All types of studies	Non-pertinent publication types (editorials, letters, reviews, conference abstracts, reports, cost-effectiveness analyses)
Publishing date	All studies published 2003 or later	All studies published before 2003
Language	English and German	All other languages

[a]Articles were excluded if no other results meeting the inclusion criteria were reported in addition to RSV detection/positivity rates
