# Supplementary material for: Burden of respiratory syncytial virus (RSV) infection in Germany: a systematic review
Source: BMC Infect Dis. 2024 Aug 20;24:844. doi: 10.1186/s12879-024-09758-3 (PMC11337829; doi:10.1186/s12879-024-09758-3)
Supplement: Supplementary file 3 — Additional file 3: Supplementary Table S3. Extracted characteristics and RSV-related outcomes of 42 included studies in detail [file 12879_2024_9758_MOESM3_ESM.pdf]

Supplementary Table S3 Extracted characteristics and RSV-related outcomes of 42 included studies in detail

Publication	Study type & data source	Population & age group	Region & time frame	RSV-related outcome measures	Results
Alchikh et al. (2019) [61]	Prospective surveillance of influenza and ARI; quality management program and Standard-of-Care	6,073 children admitted to the pediatric ED/inpatient units with ILI; median age: 1.6 years	1 pediatric hospital (Berlin); Oct. 2009 to Apr. 2015	RSV detection rate	13.2%
				Clinical manifestation (ICD-10 codes) in RSV-positive patients	Acute bronchiolitis (J21): 21.1%; acute bronchitis (J20): 29.2%; bronchitis, not specified as acute or chronic (J40): 4.5%; pneumonia, organism unspecified (J18): 18.1%; viral pneumonia, not elsewhere classified (J12): 12.2%
Ambrosch et al. (2023) [62]	Retrospective analysis	1,541 adults hospitalized with ARI and positive for RSV, Influenza A/B or SARS-CoV-2; ≥18 yrs (mean age: 73.4 yrs)	1 hospital (Regensburg); 2017 to 2020 (Jan.-Apr. each)	RSV detection rate by year	2017: 9.1%; 2018: 2.9%; 2019: 7.8%; 2020: 3.0%
				Age of RSV cases, mean	75.1 yrs
				Season peak, range over all years[a]	Cw 7 to cw 9
				Proportion of RSV cases with pneumonia as clinical manifestation[a]	32.5%
				In-hospital mortality of RSV cases[a]	11.3%
				Hospital LOS of RSV cases, mean	12.7 days
				ICU admission rate of RSV cases	13%
				Proportion of mechanically ventilated RSV cases[a]	9.5%
An der Heiden et al. (2019) [36]	Regression model; influenza virological sentinel surveillance data (weekly MAARI cases)	MAARI patients; all ages	500 primary care practices (nationwide); 2010 to 2018	Proportion of RSV infection among all MAARI	12%
				Estimated proportion of population with RSV-attributable MAARI attack rate per seasons	2010-11: 0.3%; 2011-12: 0.3%; 2012-13: 0.7%; 2013-14: 0.4%; 2014-15: 1.2%; 2015-16: 0.7%; 2016-17: 1.6%; 2017-18: 0.7%
				Age-specific RSV-attributable MAARI attack rate, median and range over all seasons	0-1 yrs: 7.5% (range: 4.0-14.8%); 2-4 yrs: 6.5% (range: 4.0-10.3%); 5-14 yrs: 0.7% (range: 0.3-1.1%)[a]; 15-34 yrs: 0.4% (range: 0.1-0.8%)[a]; 35-59 yrs: 0.5% (range: 0.1-1.5%)[a]; ≥60 yrs: 0.4% (range: 0.1-1.2%[a]
Bierbaum et al. (2014) [37]	Prospective study	369 samples from patients with ARI; all ages (median age: 6 yrs)	11 study sites; Dec. 2009 to June 2010	RSV detection rate by month	Total: 19%; Jan.: 5.5% Feb.: 25.3% Mar.: 42.7%; Apr.: 8.1%
				Season onset	Jan.
				Season peak	Mar.
				Season ending	Apr.
Cai et al. (2020a) [38]	Retrospective analysis; ICD-10 code-based surveillance systems for ALRI (primary care: SEED^{ARE}, inpatient care: ICOSARI, and national virological surveillance)	1,165 RSV-ICD cases (SEED^{ARE}), 7,345 hospitalized RSV-ICD cases (ICOSARI), 1,785 RSV-positive samples (virological surveillance); all ages	Nationwide; Oct. 2007 to Mar. 2017 (SEED^{ARE}), Jan. 2009 to Apr. 2017 (ICOSARI), Oct. 2010 to May 2017 (virological surveillance)	Proportion of RSV-ICD cases among all ARI-ICD cases (SEED^{ARE}) and all hospitalized ARI-ICD cases (ICOSARI)	0.1% (SEED^{ARE}); 0.5% (ICOSARI)
				Proportion of RSV-ICD cases aged <2 yrs among all RSV-ICD cases	66% (SEED^{ARE}); 93% (ICOSARI)
				RSV detection rate among ARI/ILI cases (virological surveillance) by age	Total: 8%; <2 yrs: 25%; 15-34 yrs: 2%; ≥60 yrs: 6%
				Season onset, range over all seasons	Mid-Oct. to end-Nov.
				Season peak, range over all seasons	End-Jan. to mid-Feb.
				Season ending, over all seasons	Mid Apr.
				Distribution of ICD-10 diagnoses among all RSV-ICD cases (SEED^{ARE})	Bronchiolitis due to RSV (J21.0): 34%; bronchitis due to RSV (J20.5): 37%; pneumonia due to RSV (J12.1): 29%
				Most frequently documented diagnosis in hospitalized RSV cases (ICOSARI)	Bronchiolitis, J21.0: (discharge diagnosis: 39%; admission diagnosis: 53%)

Publication	Study type & data source	Population & age group	Region & time frame	RSV-related outcome measures	Results
Cai et al. (2020b) [17]	Retrospective cohort study and regression analysis; ICD-10 code-based surveillance data for SARI (ICOSARI)	413,552 patients hospitalized with SARI; all ages	87 hospitals (nationwide); Jan. 2009 to May 2018	Proportion of RSV-positive SARI cases by age	Total: 2%; 0-5 mo: 43%; 6 mo-1 yr: 14%; 2-4 yrs: 3%; 5-64 yrs: 0.1%; ≥65 yrs: 0.05%
				Age distribution of RSV cases	0-5 mo: 54.6%; 6 mo-1 yr: 36.7%; 2-4 yrs: 6.0%; 5-64 yrs: 1.3%; ≥65 yrs: 1.4%
				Season onset[a] (among 0-4 yrs)	2015-16: cw 45; 2016-17: cw 45; 2017-18: cw 47
				Season peak[a] (among 0-4 yrs)	2014-15: cw 06; 2015-16: cw 05; 2016-17: cw 05; 2016-17: cw 10
				Season ending[a] (among 0-4 yrs)	2014-15: cw 19; 2015-16: cw 16; 2016-17: cw 14; 2016-17: cw 17
				Underlying conditions in RSV cases (in ICU-admitted RSV cases)	Respiratory and cardiovascular disorder specific to the perinatal period: 3.5% (12.7%); cardiovascular disease 2.5% (14%); CHD: 1.6% (7.4%); DS: 0.5% (1.9%); COPD: 0.5% (2.2%); diabetes: 0.7% (3.3%)
				Underlying conditions in deceased RSV cases	Cardiovascular disease: 60%; diabetes: 16.0%; COPD: 12.0%; CHD: 8.0%; Respiratory and cardiovascular disorder specific to the perinatal period: 4.0%; COPD: 0.0%
				Deceased RSV cases	0.3%
				Age distribution in deceased RSV cases	0-5 mo: 12%; 6 mo-1 yr: 20%; 2-4 yrs: 8%; 5-64 yrs: 12%; ≥65 yrs: 48%
				Hospital LOS of RSV cases, mean and median	5 and 6 days
				ICU admission rate of RSV cases	7%
				ICU LOS of RSV cases, mean and median	9 and 5 days
				Ventilated cases of ICU-admitted RSV cases (mean and median duration)	38% (211 and 112 hours)
Cai et al. (2022) [39]	Prospective German national ARI sentinel surveillance	33,351 RSV-positive outpatients with ARI; all ages	Nationwide; Oct. 2011 to May 2020	RSV detection rate by age[a]	Total: 8.1%; 0-4 yrs: 21%; 5-14 yrs: 3%; 15-34 yrs: 2%; 35-59: 2%; ≥60 yrs: 4%
				Season onset	2011-12: cw 1; 2012-13: cw 51; 2013-14: cw 3; 2014-15: cw 47; 2015-16: cw 50; 2016-17: cw 45; 2017-18: cw 51; 2018-19: cw 50; 2019-20: cw 51; 2021-22: cw 35
				season peak	2011-12: cw 8; 2012-13: cw 2; 2013-14: cw 6; 2014-15: cw 51; 2015-16: cw 3; 2016-17: cw 52; 2017-18: cw 4; 2018-19: cw 3; 2019-20: cw 6; 2021-22: cw 41
				Season ending	2011-12: cw 13; 2012-13: cw 12; 2013-14: cw 18; 2014-15: cw 11; 2015-16: cw 14; 2016-17: cw 10; 2017-18: cw 13; 2018-19: cw 11; 2019-20: cw 12; 2021-22: cw 50
Ehlken et al. (2005) [40]	Cost-of-illness analysis embedded in PRI.DE; economic data from patients' records or via questionnaire	115 office-based and 350 hospitalized children with CA-RSV-associated LRTI; ≤3 yrs	11 office-based pediatricians and 5 hospitals (Bochum, Dresden, Freiburg, Hamburg); 1999 to 2001	Cost per office-based RSV case, mean	Direct medical: €79; direct non-medical: €14; indirect: €70; total: €163
				Cost per hospitalized RSV case, mean	Direct medical: €2,507; direct non-medical: €105; indirect: €161; total: €2,772
				Annual economic burden due to RSV-associated LRTI (assumed incidence rate)	Office-based cases: €17.5 million (183,761 cases per year); hospitalized cases: €66 million (26,524

Publication	Study type & data source	Population & age group	Region & time frame	RSV-related outcome measures	Results
					cases per year)
Forster (2003) [41]	Prospective population-based study (PRI.DE) and hospital-based study (PID-ARI.net)	Swabs obtained of 2,099 children hospitalized with ARI (PID-ARI-net), 1,898 children hospitalized and 1,448 outpatients with LRTI (PRI.DE); ≤16 yrs (PID-ARI-net), ≤3 yrs (PRI.DE)	1 hospital (Kiel, PID-ARI-net), 11 pediatric practices and 5 pediatric hospitals (Hamburg, Dresden, Freiburg, and Bochum, PRI.DE); 1996 to 2001 (PID-ARI-net), Nov. 1999 to Oct. 2001 (PRI.DE)	RSV detection rate	9.5% (PID-ARI-net); 38% (hospitalized in PRI.DE) & 20.9% (outpatients in PRI.DE)
				Clinical manifestation in RSV-positive LRTI inpatients (PRI.DE)	LTB: 13.8%; bronchiolitis: 42.2%; bronchitis: 32.8%; pneumonia: 39.2%
				Clinical manifestation in RSV-positive LRTI outpatients (PRI.DE)	LTB: 14.1%; bronchiolitis: 32.5%; bronchitis: 22.4%; pneumonia: 32.5%
Forster et al. (2004) [42]	Prospective population-based cohort study; PRI.DE	1,487 outpatients (1,418 samples), 2,068 inpatients (1,846 samples) with acute LRTI; ≤3 yrs	11 pediatric practices and 5 pediatric hospitals (Hamburg, Dresden, Freiburg, and Bochum); Nov. 1999 to Oct. 2001	RSV detection rate in inpatients by genotype and age	RSV-A: 31.9%; RSV-B: 6.1%; 0-6 months: 55.7%; 6 months-<1 yr: 35.0%; 1-2 yrs: 27.6%; 2-3 yrs: 24.3%
				RSV detection rate in outpatients by genotype and age	RSV-A: 20.9%; RSV-B: 6.9%; 0-6 months: 36.2%; 6 months-<1 yr: 28.1%; 1-2 yrs: 25.2%; 2-3 yrs: 21.4%
				Annual incidence of RSV-related LRTI outpatient consultation	7.7 per 100 children; extrapolation for Germany: 183,761 outpatient cases
				Annual incidence of RSV-related LRTI hospitalization	1,117 per 100,000 children; extrapolation for Germany: 26,524 hospitalizations
				RSV-A vs. RSV-B detection rate in outpatients according to clinical diagnosis	LTB: 12.0% vs. 21%; bronchiolitis: 25.7% vs. 6.8%; bronchitis: 16.2% vs. 6.2%; pneumonia: 26.6% vs. 7.6%
				RSV-A vs. RSV-B detection rate in inpatients according to clinical diagnosis	LTB: 10.6% vs. 3.2%; bronchiolitis: 35.9% vs. 6.3%; bronchitis: 26.9% vs. 5.9%; pneumonia: 32.2% vs. 7.0%
				Further outpatient consultations in outpatient RSV cases, mean	2.7 visits
				Hospital LOS of inpatient RSV cases, mean	7 days
				Consultations after discharge in inpatient RSV cases, mean	3.6 visits
Gröhndal et al. (2014) [43]	Prospective study; PID-ARI.net	3,998 samples from children hospitalized with ALRI; ≤18 yrs	Pediatric hospitals (Mainz, Wiesbaden and Kiel); Jul. 2009 to Mar. 2011	RSV detection rate of all specimens (among all positive samples)	13.3%
				Season onset[a]	2009-10: cw 50; 2010-11: cw 44
				Season peak[a]	2009-10: cw 10; 2010-11: cw 1
				Season ending[a]	2009-10: cw 17; 2010-11: cw 12
Hartmann et al. (2022) [19]	Retrospective study; medical record review	312 children hospitalized with RSV infection; ≤5 yrs	1 hospital (Würzburg); 2015 to 2018	Age distribution of RSV cases	0-<6 months: 35%; 6-<12 months: 15%; 1-<2 years 25%; 2-5 years: 25%
				Most frequent co-infections	Any co-infection: 29.8%; rhinovirus: 15.1%; coronavirus: 6.4%; adenovirus: 6.4%
				Bronchiolitis as clinical manifestation by age	Total: 62.8%; <6mo: 79.6%, 6-<12 mo: 81.3%, 1-<2 yrs: 51.3%, 2-5 yrs: 39.7%
				Pneumonia as clinical manifestation by age	Total: 29.5%; <6mo: 19.4%, 6-<12 mo: 18.8%, 1-<2 yrs: 29.5%, 2-5 yrs: 50.0%
				Most frequently underlying conditions	Premature birth among infants <1 yr: 16.7%; CHD: 8.0%; BPD: 2.6%; NMI: 2.2%; immunodeficiency: 1.6%; Down syndrome: 0.6%

Publication	Study type & data source	Population & age group	Region & time frame	RSV-related outcome measures	Results
				Proportion of deceased RSV cases by age	Total: 0.3%; <6mo: 0.0%; 6-<12 mo: 0.0%, 1-<2 yrs: 0.0%, 2-5 yrs: 1.3%
				Setting before hospitalization	GP: 63%; ED: 34.9%; outpatient clinic: 3.2%
				Hospital LOS, median	5.0 days
				ICU admission rate by age	Total: 5.1%; <6mo: 9.3%; 6-<12 mo: 0.0%, 1-<2 yrs: 3.8%, 2-5 yrs: 3.8%
				Oxygen treatment by age	Total: 57.7%; <6mo: 62.0%; 6-<12 mo: 56.3%, 1-<2 yrs: 51.3%, 2-5 yrs: 59.0%
				Treatment by method	Antibiotics: 43.6%; HFNC: 3.5%; invasive ventilation: 1.9%; non-invasive ventilation: 1.0%
Hönemann et al. (2023) [63]	Prospective sampling and retrospective data retrieval from patient charts	4,549 (<18 years) and 8,042 (≥18 years) in- and outpatients (19,734 samples in total); All ages	1 hospital (Leipzig); Oct. 2017 to Sep. 2022	RSV detection rate by season	Total: 7.1%; 2017-18: 6.4%; 2018-19: 8.3%; 2019-20: 4.0%; 2021-22: 11.5%
				RSV detection rate by age and season (pediatric vs adult)	2017-18: 25.6 vs. 3.8%; 2018-19: 30.7 vs. 6.8%; 2019-20: 27.7 vs. 3.8%; 2021-22: 48.9 vs. 9.4%
				Age of RSV cases, mean	Children: 1.3 yrs; adults: 62.4 yrs
				Proportion of pediatric RSV-positive cases among all RSV-positive cases by season	Total: 75.4%; 2017-18: 71.4%; 2018-19: 68.3%; 2019-20: 69.5%; 2021-22: 88.9%
				Season onset	2017-18: cw 41; 2018-19: cw 47; 2019-20: cw 45; 2021-22: cw 27
				Season peak	2017-18: cw 6; 2018-19: cw 6; 2019-20: cw 7; 2021-22: cw 40
				Season ending	2017-18: cw 22; 2018-19: cw 22; 2019-20: cw 14; 2021-22: cw 9
				Clinical manifestation in pediatric RSV cases	LRTI: 82.4%; bronchiolitis/bronchitis: 67.1%; pneumonia: 19.0%
				Clinical manifestation in adult RSV cases	LRTI: 50.8%; bronchitis: 8.4%; pneumonia: 34.2%
				Underlying conditions in pediatric RSV cases	Premature birth: 19.6%; immunosuppression: 2.0%; cardiac failure: 1.8%; asthma: 1.5%; cardiovascular disease: 0.8%; diabetes: 0.7%; COPD: 0.0%
				Underlying conditions in adult RSV cases	Cardiovascular disease: 58.8%; immunosuppression: 38.0%; diabetes: 31.2%; cardiac failure: 17.7%; COPD: 17.1%; asthma: 5.7%
				Proportion of RSV-A in all RSV-positive cases by season	Total: 52.1%; 2017-18: 24.9%; 2018-19: 59.7%; 2019-20: 77.3%; 2021-22: 56.0%
				Proportion of pediatric vs. adult inpatients	95.8% vs. 78.9%
				In-hospital mortality of pediatric vs. adult RSV cases	0.0% vs. 6.1%
				Hospital LOS of pediatric vs. adult RSV cases, mean	6.5 days vs. 18.3 days
				ICU admission rate of pediatric vs. adult RSV cases	10.5% vs. 25.0%
				ICU LOS of pediatric vs. adult RSV cases, mean	8.8 vs. 7.6 days
				Treatment of pediatric RSV cases	Inhalation: 82.9%; oxygen: 59.1%; ventilation: 1.8%
				Treatment of adult RSV cases	Inhalation: 18.2%; oxygen: 23.3%; ventilation: 8.1%

Publication	Study type & data source	Population & age group	Region & time frame	RSV-related outcome measures	Results
Kiefer et al. (2023) [44]	Retrospective analysis	1,903 patients hospitalized with RSV (1,446 children, 457 adults); all ages (median age of children: 8 mo; adults: 77 yrs)	2 hospitals (Regensburg); 2016 to 2023	Season onset (pediatric population)[a]	2016-17: cw 1; 2017-18: cw 51; 2018-19: cw 46; 2019-20: cw 46; 2021-22: cw 31; 2022-23: cw 42
				Season peak (pediatric population)[a]	2016-17: cw 6; 2017-18: cw 9; 2018-19: cw 5; 2019-20: cw 5; 2021-22: cw 43; 2022-23: cw 47
				Season ending (pediatric population)[a]	2016-17: cw 14; 2017-18: cw 19; 2018-19: cw 16; 2019-20: cw 15; 2021-22: cw 50; 2022-23: cw 11
				Underlying conditions in pediatric ICU cases	Premature birth: 23.1%; CHD: 7.7%; no comorbidities: 51.9%
				Underlying conditions in adult cases	Cardiovascular disease: 53.6%; diabetes: 30.4%; COPD: 28.7%
				Hospital LOS pediatric vs. adult cases, median	4 vs. 8 days
				ICU admission rate of pediatric vs. adult cases	3.6% vs. 10.1%
Liese et al. (2003) [45]	Population-based cohort study; questionnaire to parents of preterms after NICU discharge (after birth)	717 preterm infants admitted to NICU (wGA <35); ≤18 mo	9 NICUs in Southern Germany; Nov. 1998 to Oct. 1999; questionnaires send out in Aug. and Nov. 2000	Hospitalization due to RSV-related ARI after NICU discharge	5.2% (laboratory-proven: 1.8%; probable: 3.4%)
				Age of RSV-related hospitalization, mean	7.4 mo
				Incidence rate of RSV-related hospitalization	69.8 cases per 1000 preterm infants
				Clinical manifestation among hospitalized RSV cases	Pneumonia: 27%; obstructive bronchitis/bronchiolitis/acute bronchitis: 67.6%
				Underlying conditions among hospitalized RSV cases	Cardiac abnormalities: 32.4%; CLD: 21.6%
				Hospital LOS of hospitalized RSV cases, median	8 days
				Treatment of hospitalized RSV cases	Inhalation: 91.9%; mechanical ventilation: 37.8%; antibiotics: 62.2%
Maison et al. (2022) [64]	Retrospective analysis	3,086 children from hospital outpatient and inpatient facilities with confirmed viral respiratory or gastrointestinal infection; ≤18 yrs	1 hospital (Munich): Jan. 2017 to Oct. 2021	RSV detection rate by year	2017: 46%; 2018: 30%; 2019: 31%; 2020: 15%; 2021: 26%
				Season onset[a]	2017-18: Nov.; 2018-19: Oct.; 2019-20: Dec.; 2021-22: Jul.
				Season peak[a]	2017-18: Feb.; 2018-19: Feb.; 2019-20: Jan.
				Season ending[a]	2017-18: May; 2018-19: Apr.; 2019-20: Apr.
Mentel et al. (2005) [65]	Observational study	330 children hospitalized with suspected viral respiratory illness (356 samples)	1 pediatric hospital (Greifswald); Jan. 2000 to May 2001	RSV detection rate	26.4%
				Type distribution in RSV-positive samples	RSV-A: 86.8%; RSV-B: 9.2%
				Proportion of severe RSV infections (oxygen or assisted ventilation)	72.3%
				Age distribution of severe RSV infections	0-6 mo: 32%; 7-12 mo: 26%; >1 yr: 42%
Meyer et al. (2022) [66]	Retrospective cohort analysis	748 children with respiratory symptoms; ≤4 yrs	1 hospital (Cologne); Mar. 2020 to Nov. 2021	RSV detection rate	22.6%
				Hospital LOS of RSV cases, median	4 days
				ICU admission rate of RSV cases	10.1%
				Treatment of RSV cases	Oxygen: 39.6%; HFNC: 24.3%; CPAP: 1.8%; IMV: 1.2%
Reiche & Schweiger (2009) [46]	Prospective epidemiological study	6,115 samples from patients with respiratory illness; all ages	2 hospitals and ~150 medical practices (nationwide); Oct. 1998 to Sep. 2007	RSV detection rate by age	Total 23%; <3 mo: 51%; 3-6 mo: 47%; 7-12 mo: 34%; 1-3 yrs: 23%; >3yrs: 7%; unknown age: 28%
				Season onset	1999-2000: cw 52; 2000-01: cw 49; 2001-02: cw 47; 2002-03: cw 42; 2003-04: cw 1; 2004-05: cw 47; 2005-06: cw 7; 2006-07: cw 50

Publication	Study type & data source	Population & age group	Region & time frame	RSV-related outcome measures	Results
				Season peak	1999-2000: cw 4; 2000-01: cw 8; 2001-02: cw 13; 2002-03: cw 51; 2003-04: cw 12; 2004-05: cw 7; 2005-06: cw 15; 2006-07: cw 8
				Season ending	1999-2000: cw 17; 2000-01: cw 17; 2001-02: cw 30; 2002-03: cw 14; 2003-04: cw 18; 2004-05: cw 14; 2005-06: cw 19; 2006-07: cw 17
				Proportion of RSV-A among all differentiated RSV-positive samples by season	1998-99: 20%; 1999-2000: 93%; 2000-01: 58%; 2001-02: 67%; 2002-03: 34%; 2003-04: 75.4%; 2004-05: 80.1%; 2005-06: 64.1%; 2006-07: 52.6%
Schreiner et al. (2019) [67]	Retrospective cohort analysis	2,464 children hospitalized with ALRI and positive for at least one pathogen; median age: 18 mo	1 hospital (Mainz): Jan. 2008 to Jan. 2013	RSV detection rate	24.0%
				Age of RSV-infected children, median	9 mo
				Season peak	2007-08: Mar.; 2008-09: Dec.; 2009-10: Mar.; 2010-11: Jan.; 2011-12: Feb.
				Hospital LOS of RSV cases	7 days
				Treatment of RSV cases	Inhalation: 73.8%; antibiotics: 44.4%
Simon et al. (2007) [47]	Prospective surveillance study; German database for the inpatient management of RSV infections in pediatrics (DSM RSV Ped)	406 preterm and 1,162 term children hospitalized with RSV and treated for at least 24h[b]	14 pediatric hospitals (nationwide); 1999 to 2005	Gestational age, median	Preterms: 33 weeks; terms: 39 weeks
				Underlying conditions in preterm vs. term children	CHD: 17.2% vs. 5.2%; NMI: 7.4% vs. 3.7%; ≥2 risk factors: 7.9% vs. 0.6%
				RSV-attributable mortality in preterm vs. term children	1.2% vs. 0.2%
				Hospital LOS in preterm vs. term children, median	8 days vs. 6 days
				ICU admission rate in preterm vs. term children	23% vs. 8%
				Treatment in preterm vs. term children	Oxygen: 67% vs. 51%; antibiotics: 51% vs. 35%
Simon et al. (2008) [48]	Prospective surveillance study; German database for the inpatient management of RSV infections in pediatrics (DSM RSV Ped)	1,478 children hospitalized with CA-RSV[b]	14 pediatric hospitals (nationwide); Nov. 1999 to Apr. 2005	Age distribution	1-28 days: 7%; 5 wks-5 mo: 49%; 6-12 mo: 23%; 13-24 mo: 11%; >24 mo: 10%
				RSV-attributable mortality	0.4% (all-cause mortality: 0.5%)
				RSV-positive days, median	7 days
				Hospital LOS, median	7 days
				ICU admission rate	9%
				Treatment	Oxygen: 54%; antibiotics: 38%
				Underlying conditions	Premature birth: 24.1%; mechanical ventilation in medical history: 12.6%; CHD: 7.3%; NMI: 3.9%; CLD: 2.4%; very low birthweight (≤1499 g): 6.6%; extremely low birth weight (≤999 g): 3.0%; patients with ≥2 risk factors: 6.9%
Simon et al. (2011) [49]	Prospective single-arm observational study; German Synagis Registry	9,833 palivizumab-immunized children hospitalized for any reason; <2 yrs	Nationwide; 2002 to 2007	RSV-related hospitalization	2.5% (worst-case scenario); 1.6% (lab-proven)
				Underlying conditions among RSV-positive cases	Premature birth: 88%; BPD: 47%; CHD: 27%
				ICU admission rate among RSV-positive cases	37%
				Treatment of RSV-positive cases	Oxygen: 69%; mechanical ventilation: 10%
Simon et al. (2018a) [50]	Prospective single-arm observational study; German Synagis Registry	12,729 children from hospital outpatient and inpatient facilities who received palivizumab in	Nationwide; Sep. 2009 to June 2016	RSV-related hospitalization	0.7%; 0.8% in CHD patients
				Clinical manifestation in RSV-related hospitalizations	Bronchiolitis: 37.6%; bronchitis: 28.2%; pneumonia: 32.9%
				Underlying conditions among hospitalized	Premature birth: 78.8%; CLD/BPD: 41.2%; CHD:

Publication	Study type & data source	Population & age group	Region & time frame	RSV-related outcome measures	Results
		their first season[c];<br><25 mo		patients (incl. non-RSV-related hospitalizations)	34.2%; DS: 3.8%
				ICU admission rate in hospitalized RSV cases (median duration)	16.9% (3 days)
				Mechanical ventilation rate in hospitalized RSV cases (median duration)	8% (2.5 days)
Simon et al. (2018b) [51]	Prospective single-arm observational study; German Synagis Registry	920 children from hospital outpatient and inpatient facilities who received palivizumab in 2 consecutive seasons[c];<br><25 mo	Nationwide;<br>Sep. 2009 to June 2016	RSV-related hospitalization	0.9%
				Clinical manifestation in RSV-related hospitalizations	Bronchiolitis: 18%; bronchitis: 27%; pneumonia:27%
Simon et al. (2018c) [52]	Prospective single-arm observational study; German Synagis Registry	249 palivizumab-immunized children with DS from hospital outpatient and inpatient facilities[c];<br><25 mo	Nationwide;<br>Sep. 2009 to June 2016	RSV-related hospitalization in patients with vs. without DS	1.2% vs. 0.7%
				Underlying condition for complicated RSV infection in patients with DS vs without DS	Premature birth (wGA <36): 27.3% vs. 85.9%; CHD: 85.1% vs. 26.2%; NMI: 38.3% vs. 8.7%; immunodeficiency: 11.3% vs. 1.2%
Streng et al. (2019) [53]	Surveillance study; case report forms and hospital patient files	603 children with RSV-RTI from three settings:<br>1–5 yrs (pediatric practices (PP)),<br>≤16 yrs (pediatric hospital ward (PW)),<br>1 mo-16 yrs (PICU)	33 PP, 1 PW and 9-23 PICU (Bavaria);<br>2013 to 2015 (PP),<br>Jan. 2012 to Mar. 2017 (PW),<br>Dec. 2010 to May 2013 (9 PICU) and 2014 to 2017 (23 PICU)	Clinical manifestation per setting	Bronchitis/Bronchiolitis: 41.7% (PP); 46.7% (PW); 80.8% (PICU); pneumonia: 6.9% (PP); 28.5% (PW); 69.2% (PICU)
				Underlying condition for RSV-A infection per setting (PP vs. PW vs. PICU)	Premature birth: 4.2% vs. 12.7% vs. 25.0%
				Genotype distribution	RSV-A: 56.6%; RSV-B: 39.0%; RSV-A and RSV-B co-infection: 0.2%; no identified genotype: 4.3%
				Hospital LOS of children with RSV-A per setting (PW vs. PICU), median	3.2 vs. 7.5 days
				PICU LOS of children with RSV-A, median	4.5 days
				Treatment of children with RSV-A per setting (PW vs. PICU)	Oxygen: 43.0% vs. 89.4% (median duration: 2.8 vs. 4.9 days)
				Treatment of children with RSV-A in PICU	CPAP: 34.6% (median duration: 2.5 days); mechanical ventilation: 10.6% (median duration: 6.2 days)
				Treatment of children with RSV-A in PICU stratified by premature birth status (preterm vs. term)	Median hospital LOS: 8.2 vs. 7.4 days; median PICU LOS: 4.9 vs. 4.4 days; Oxygen: 88.5 vs. 89.7% (median duration: 5.3 vs. 4.8 days); CPAP: 50.0 vs. 29.5% (median duration: 2.7 vs. 2.6 days)
Tabatabai et al. (2014) [68]	Retrospective analysis; medical records	242 samples from children hospitalized with URTI or LRTI symptoms;<br>≤2 yrs (mean age: 7.9 mo)	Pediatric department at 1 hospital (Heidelberg);<br>Oct. 2012 to Apr. 2013	RSV detection rate	55.4%
				Age distribution of RSV-positive children	0-6 mo: 62.7%; >6-12 mo: 14.9%; >12-18 mo: 14.9%; >18-24mo: 7.5%; mean age: 6.5 mo
				Season onset[a]	Cw 48
				Season peak[a]	Cw 9
				Clinical manifestation in RSV-positive patients	Bronchitis/bronchiolitis: 75.4%; pneumonia: 17.2%
				Genotype distribution in RSV-positive patients	RSV-A: 82.1%; RSV-B: 17.9%
				Hospital LOS of RSV cases, mean	5.3 days
				ICU admission rate of RSV cases	4.5%

Publication	Study type & data source	Population & age group	Region & time frame	RSV-related outcome measures	Results
Tabatabai et al. (2022) [33]	Prospective cohort analysis; questionnaire,	946 samples from children hospitalized with ARI[d]; ≤18 yrs (mean age: 9.5 mo)	Pediatric department at 1 hospital (Heidelberg); Oct. 2014 to Apr. 2017	RSV detection rate	42.8%
				Age distribution of RSV-positive children	≤6 mo: 56.3%; 6 mo-<2 yrs: 28.1%; 2-<5 yrs: 13.3%; ≥5 yrs: 2.2%; mean age: 0.39 yrs
				Season onset[a]	2014-15: cw 46; 2015-16: cw 47; 2016-17: cw 47
				Season peak[a]	2014-15: cw 52; 2015-16: cw 4; 2016-17: cw 5
				Season ending[a]	2014-15: cw 14; 2015-16: cw 12; 2016-17: cw 12
				Clinical manifestation in RSV-positive children	Bronchiolitis: 36.0%; bronchitis: 28.1%; pneumonia: 14.6%
				Hospital LOS, median	3 days
Tabatabai et al. (2023) [34]	Prospective cohort analysis; questionnaire	1,353 children hospitalized with ARI[d]; ≤18 yrs (median age: 10.5 months)	Pediatric department at 1 hospital (Heidelberg); Nov. 2014 to Apr. 2018	RSV detection rate by season[a]	Total: 42.1%; 2014-15: 51.4%; 2015-16: 32.4%; 2016-17: 46.1%; 2017-18: 40.5%
				Proportion of RSV-positive children among all ARI cases by age[a]	≤6 mo: 57.6%; 6 mo -<2 yrs: 35.5%; 2-<5 yrs: 27.9%; ≥5 yrs: 20.7%; median age: 5 months
				Season onset[a]	2017-18: cw 49
				Season peak[a]	2017-18: cw 8
				Season ending[a]	2017-18: cw 15
				Clinical manifestation in RSV-monoinfected children	Bronchiolitis/bronchitis: 63.9%; pneumonia: 14.3%
				Most frequent co-infection	RSV/rhinovirus: 27.2%; RSV/coronovirus: 18.6%
Tenenbaum et al. (2022) [54]	Prospective case-cohort analysis; Clinician-Led Reporting System (CLRS)	Hospitalized RSV cases; ≤5 yrs	Pediatric hospitals (nationwide); Oct. 2021 to Mar. 2022	Age distribution	<1 yr: 66%; 1yr: 15%; 2 yrs: 10%; 3yrs: 5%; 4 yrs: 2%; 5 yrs: 1%
				Season peak	Late Oct./early Nov.
				Cases per hospital per day between Oct.-Jan., mean (range)	General wards: 3.6 (0.3-8); ICU admissions: 0.4 (0-0.9)
				Proportion of ICU-admitted patients within hospitalized group by month	Total: 8.7%; Oct: 8.3%; Nov: 8.3%; Dec: 9.4%; Jan: 15.8%; Feb:14.3%; Mar.: 33.3%
				Proportion of hospitalized patients requiring ventilation	Ventilation: 1.3%; non-invasive respiratory support: 10.2%
Terletskaia-Ladwig et al. (2005) [55]	Prospective epidemiological study	3,577 samples from children with suspected RSV infection; ≤18 yrs	Pediatric hospitals and practitioners (Southern Germany); Jan. 1996 to June 2004	RSV detection rate	32%
				Age distribution of RSV cases	<3 mo: 40%; 3-6 mo: 30%; 7-12 mo: 20%; 1-3 yrs: 10%; >3 yrs: <1%
				Season onset	1995-96: cw 1; 1996-97: cw 49; 1997-98: cw 2; 1998-99: cw 48; 1999-2000: cw 50; 2000-01: cw 48; 2001-02: cw 6; 2002-03: cw 45; 2003-04: cw 1
				Season peak	1995-96: cw 12; 1996-97: cw 8; 1997-98: cw 13; 1998-99: cw 5; 1999-2000: cw 14; 2000-01: cw 6; 2001-02: cw 20; 2002-03: cw 51; 2003-04: cw 14
				Season endin	1995-96: cw 17; 1996-97: cw 13; 1997-98: cw 20; 1998-99: cw 18; 1999-2000: cw 19; 2000-01: cw 15; 2001-02: cw 23; 2002-03: cw 6; 2003-04: cw 19
Topoulos et al. (2019) [69]	Retrospective cohort analysis	214 in- and outpatient with ARI and confirmed influenza virus and/or RSV infection;	1 hospital (Bochum); Jan. to Apr.2018	RSV detection rate	14%
				Season peak	Cw 9
				Proportion of pneumonia among RSV-positive patients	50%

Publication	Study type & data source	Population & age group	Region & time frame	RSV-related outcome measures	Results
		≥18 yrs (mean age: 69.9 yrs)		Proportion of fatal cases among RSV-positive pneumonia patients	13.3%
Vogel et al. (2016) [70]	Prospective study; questionnaire, medical records	272 children hospitalized with symptoms of ARI and/or ILI; ≤18 yrs (median age: 7 mo)	1 pediatric hospital (Duesseldorf); Nov. 2009 to Apr. 2010	RSV detection rate	32.0% (monoinfection: 78.2%; co-infection: 21.8%)
				Median age of RSV-positive children	7 mo
				Season peak	Mar.
				Clinical manifestation	Pneumonia: 53%
				Genotype distribution in RSV-positive samples	RSV-A: 64.4%; RSV-B: 35.6%
				Hospital LOS, median	4 days
Wasem et al. (2008) [71]	Analysis of data from 2 prospective surveillance studies; PRI.DE and PID-ARI.net	1,054 children: 461 cases (PRI.DE), 593 cases (PID-ARI.net) hospitalized with LRTI; ≤36 mo	1 hospital (Freiburg); PRI.DE study: 1999 to 2001, PID-ARI.net: Oct. 2002 to June 2005	RSV detection rate (by study and age group)	PRI.de: 39.5%; PID-ARI.net: 29.8%; 0-12 months: 42.8%; 13-24 mo: 23.4%; 25-36 mo: 23.1%
				Diagnosis for RSV-related hospitalizations	Pneumonia: 61.3%; bronchiolitis: 30.1%; bronchitis. 5.6%; LTB: 3.1%
Weigl et al. (2003) [32]	Retrospective nested case-control study; questionnaire	1,316 children hospitalized with LRTI[e]; <2 yrs	3 pediatric hospitals (Kiel and Flensburg); Jul. 1996 to June 2000	RSV detection rate	16.5%
				Median age	128 days
				Clinical manifestation	Pneumonia: 55.8%; wheezing bronchitis: 33.6%; bronchitis: 10.6%; LTB: 0.0%
Weigl et al. (2004) [35]	Retrospective nested case-control study; questionnaire	1,316 children hospitalized with LRTI[e]; <2 yrs	3 pediatric hospitals (Kiel and Flensburg); Jul. 1996 to June 2000	Underlying conditions in RSV-positive children	Premature birth: 19.4%; BPD: 1.4%; other pulmonary: 2.8%; cardiological: 3.2%; no comorbidity: 89.4%
				Hospital LOS of RSV cases, median	9 days
				ICU admission rate of RSV cases	5.1%
				Treatment of RSV cases	Antibiotic (>3 days): 51.2%; oxygen (>1 day): 37.8%; mechanical ventilation: 2.8%
Weigl et al. (2005) [56]	Prospective cross-sectional study	514 children hospitalized with CAP; ≤16 yrs	2 hospitals (Kiel); Jul. 1996 to June 2000	RSV detection rate by age (based on PCR of 250 CAP cases)	Total: 25.2%; <1 yr: 51.9%; 1-4 yrs: 18.9%; 5-16 yrs: 1.6%
				Incidence of RSV-related hospitalized CAP (extrapolated for all of Germany)	7,240 cases per year
Weigl et al. (2007) [57]	Retrospective sentinel-type analysis; PID-ARI.net	18,899 samples from children with LRTI or complicated upper ARI patients (86.9% inpatient and 13.1% outpatient); <16 yrs (median age: 22 mo)	8 hospitals and pediatric offices (Kiel, Freiburg and Mainz); Jul.1996 to June 2006	RSV detection rate by season within all samples	1996-97: 20.0%; 1997-98: 10.9%; 1998-99: 13.4%; 1999-2000: 13.3%; 2000-01: 26.7%; 2001-02: 10.8%; 2002-03: 15.3%; 2003-04: 11.0%; 2004-05: 17.0%; 2005-06: 10.1%
				Median age of RSV- positive cases[a]	Inpatients: 7 mo; outpatients: 24 mo
				Season onset[a]	1996-97: Dec.; 1997-98: Dec.; 1998-99: Sep.; 1999-2000: Dec.; 2000-01: Oct.; 2001-02: Dec.; 2002-03: Oct.; 2003-04: Dec.; 2004-05: Oct.; 2005-06: Nov.
				Season peak[a]	1996-97: Feb.; 1997-98: Mar.; 1998-99: Dec.; 1999-2000: Mar.; 2000-01: Jan.; 2001-02: Apr.; 2002-03: Dec.; 2003-04: Mar.; 2004-05: Jan.; 2005-06: Mar.
				Season ending[a]	1996-97: May; 1997-98: Jul.; 1998-99: May; 1999-2000: Jun.; 2000-01: May; 2001-02: Jul.; 2002-03: Apr.; 2003-04: Jun.; 2004-05: May; 2005-06: May

Publication	Study type & data source	Population & age group	Region & time frame	RSV-related outcome measures	Results
Weinberger et al. (2018) [58]	Prospective sentinel study; questionnaire	965 adult outpatients with long-lasting cough (≥7 days); ≥18 yrs	35 GPs (Krefeld and Rostock); Oct. 2001 to Dec. 2004	Proportion of patients with positive RSV serology	7.6%
				Medical resource use (based on 49 RSV patients)	Mean number of visits: 4.3; referred to specialist: 35.0%; any prescription: 89.8%; antibiotics: 73.4%
Wetzke et al. (2023) [59]	Prospective observational study; pedCAPNETZ	437 children with outpatient-treated or hospitalized CAP; <18 yrs (median age 4.5 yrs)	8 study centers across Germany; Dec. 2014 to Aug. 2020	RSV detection rate by age[a]	Total: 20.3% (monoinfection: 11.7%; co-infection: 8.6%); 0-<2 yrs: 29.8%; 2-<6 yrs: 16.8%; 6-<12 yrs: 1.9%; 12-<18 yrs: 0.0%
				Detection rate of RSV cases in outpatient vs. inpatient	Total: 17.6 vs. 22.5%; <6 yrs: 28.3 vs. 29.5%; 6-<18 yrs: 0.0 vs. 1.9%
Wilkesmann et al. (2007) [60]	Prospective surveillance study; German database for the inpatient management of RSV infections in pediatrics (DSM RSV Ped)	1,541 children with RSV infection (incl. 70 patients with neuromuscular impairment (NMI))	14 pediatric hospitals; 1999 to 2005	Age distribution of hospitalized children without NMI	1-28 days: 6%; 5 wks-5 mo: 44%; 6-12 mo: 30%; 13-24 mo: 11%; >24 mo: 9%; median age: 145 days
				Age distribution of hospitalized children with NMI	1-28 days: 4%; 5 wks-5 mo: 18%; 6-12 mo: 22%; 13-24 mo: 23%; >24 mo: 33%; median age: 430 days
				Underlying conditions of children hospitalized with NMI vs. without NMI	WGA, median: 38 vs. 39 weeks; premature birth: 41.1 vs. 25.2%; mechanical ventilation in medical history: 65.8 vs. 12.6%; CHD: 26.0 vs. 7.5%; CLD: 8.2 vs. 2.9%; very low birthweight (<1499g): 17.8 vs. 7.8%; extremely low birthweight (<999g): 9.6 vs. 3.7%
				Mortality with NMI vs. without NMI	5.5 vs. 0.2%
				Hospital LOS with NMI vs. without NMI, median	11 vs. 7 days
				ICU admission rate of children with NMI vs. without NMI	45% vs. 10%
				Treatment of children hospitalized with NMI vs. without NMI	Oxygen: 49% vs. 52%; ventilation: 9.6% vs 1.9%

ALRI: acute lower respiratory tract infection; BPD: bronchopulmonary disease; CA(P): community-acquired (pneumonia); CHD: congenital heart disease; CLD: chronic lung disease; CPAP: continuous positive airway pressure; cw: calendar week; DS: down syndrome; GP: general practitioner; HFNC: high-flow nasal cannula; ICD-10: 10th revision of the International Classification of Diseases; ICU: intensive care unit; ILI: influenza-like illness; IMV: intermittent mandatory ventilation; LOS: length of stay; LRTI: lower respiratory tract infection; LTB: Laryngotracheo-bronchitis (equals Croup); MAARI: medically attended acute respiratory infection; mo: months; NICU: neonatal intensive care unit; NMI: neuromuscular impairment; OR: odds ratio; OSP: one season population; PICU: pediatric intensive care unit; PID.ARI.net: pediatric infectious diseases network on acute respiratory tract infections; PP: pediatric practice; PRI.DE: Paediatric Respiratory Infection in Germany; PW: pediatric hospital ward; RSV: respiratory syncytial virus; (S)ARI: (severe) acute respiratory infections; SSP: second season population; URTI: upper respiratory tract infection; wGA: gestational age in weeks; wks: weeks; yrs: years.

Numbers in square brackets refer to references in main text.

[a] Graphically shown only (in case of manual readout from graphs regarding seasonality, the onset and ending was determined by the first or last calendar week or month with no gaps of RSV cases in between)

[b] Partially overlapping population in Simon 2007 and Simon 2008

[c] Partially overlapping population in Simon 2018a, Simon 2018b and Simon 2018c

[d] Partially overlapping population in Tabatabai 2022 and Tabatabai 2023, same outcomes were not extracted repeatedly

[e] Identical populations of Weigl 2003 and Weigl 2004, same outcomes were not extracted repeatedly
